# Recent Progress for Single-Molecule Magnets Based on Rare Earth Elements

**DOI:** 10.3390/ma16093568

**Published:** 2023-05-06

**Authors:** Xiang Yin, Li Deng, Liuxia Ruan, Yanzhao Wu, Feifei Luo, Gaowu Qin, Xiaoli Han, Xianmin Zhang

**Affiliations:** 1 Key Laboratory for Anisotropy and Texture of Materials (Ministry of Education), School of Material Science and Engineering, Northeastern University, Shenyang 110819, China; 2 Research Center for Humanoid Sensing, Zhejiang Laboratory, Hangzhou 311100, China; 3 Taian Weiye Electromechanical Technology Co., Ltd., Taian 271000, China

**Keywords:** single-molecule magnets, rare earth elements, phthalocyanines, magnetism, preparation technology

## Abstract

Single-molecule magnets (SMMs) have attracted much attention due to their potential applications in molecular spintronic devices. Rare earth SMMs are considered to be the most promising for application owing to their large magnetic moment and strong magnetic anisotropy. In this review, the recent progress in rare earth SMMs represented by mononuclear and dinuclear complexes is highlighted, especially for the modulation of magnetic anisotropy, effective energy barrier (U_eff_) and blocking temperature (T_B_). The terbium- and dysprosium-based SMMs have a U_eff_ of 1541 cm^−1^ and an increased T_B_ of 80 K. They break the boiling point temperature of liquid nitrogen. The development of the preparation technology of rare earth element SMMs is also summarized in an overview. This review has important implications and insights for the design and research of Ln-SMMs.

## 1. Introduction

Single-molecule magnets (SMMs), with the slow relaxation of magnetization and quantum tunneling [1,2,3], are considered a significant discovery in the field of nanomagnetism [4]. SMMs are often used to fabricate nanoscale devices and high-density data storage media [5,6,7,8,9]. Notably, Mn_12_ [1] and Fe cluster [10] are the earlier discovered SMMs, which belong to 3*d* SMMs.

Since 2003, the introduction of lanthanide rare earth ions has allowed SMMs to enter a new stage. Rare earth SMMs exhibit magnetic bistability at a higher blocking temperature (T_B_) than 3*d* SMMs because lanthanide ions are *f*-orbital-based elemental ions with unparalleled single-ion anisotropy, T_B_ is a key performance parameter of an SMM, one description of which refers the maximum temperature at which it is possible to observe hysteresis in the field-dependence of the magnetization, subject to the field sweep rate. Meanwhile, phthalocyanines (Pcs) are large rings with 18π electron conjugation and have a wide range of applications in spintronics. Therefore, LnPc_2_ SMMs have shown great potential for spintronics and device applications. The first example of the [TbPc_2_]^−^ effective energy barrier (U_eff_, that is the potential energy required for molecular magnetization (or magnetic moment) reversal) of 331 cm^−1^ broke the record of U_eff_ for multinuclear 3*d* SMMs [11]. Subsequently, scientists have shown great interest in studying not only mononuclear rare earth SMMs but also binuclear rare earth SMMs and multinuclear rare earth SMMs [12,13].

This paper reviews the main progress of rare earth SMM research in the last 20 years, especially Tb/Dy-Pc_2_ SMMs. At the same time, we elaborate on the techniques of SMM preparation and how to regulate their properties.

## 2. Phthalocyanine Single-Molecule Magnets

SMMs are nanosized molecules with a stable magnetization intensity coming from within a single molecule and therefore can be used as independent magnetic functional units. In essence, a maximum value of the imaginary part of the magnetization related to the external field frequency occurs when Alternating Current magnetization rate tests are performed at low temperatures [1,14]. The development stages of monomolecular magnets are as follows. First, are transition metal monomolecular SMMs (Mn_12_ and Fe clusters); then are rare earth single-molecule magnets, mainly lanthanide-based metal SMMs. Therefore, the use of rare earth metal ions, especially Tb and Dy ions, to construct SMMs is still an effective method to improve the performance of SMMs [15].

The 4*f* orbitals of lanthanide ions are inner orbitals and thus have strong spin-orbit coupling, which allows the crystal field interaction to be regarded as perturbative. Therefore, Ln-SMMs (Ln = Tb, Dy) have become an important part of the field of SMMs, are widely favored by researchers and have been reported far more than other metallic SMMs, occupying half of the field of molecular magnets.

**Figure 1:**
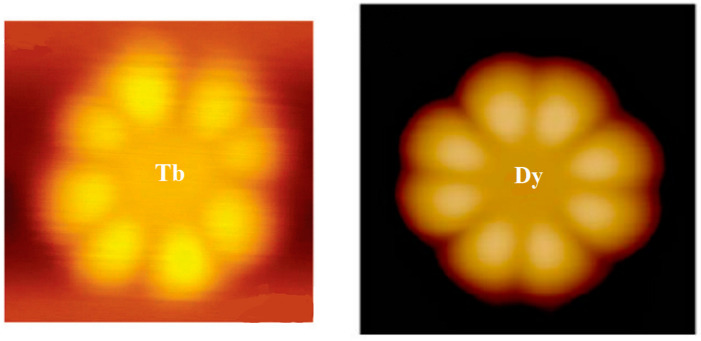
STM images in experiments. (**Left**): TbPc_2,_ bars indicate 1 nm; (**Right**): DyPc_2,_image sizes: 3 × 3 nm^2^ [16,17].

From Figure 1, we can see that the scanning tunneling microscopy (STM) images of LnPc_2_ (Ln = Tb, Dy) molecules observed by the experiment have the shape of eight lobes. However, DyPc_2_ is more regular than TbPc_2_ [16,17].

### 2.1. Structure and Category

**Figure 2:**
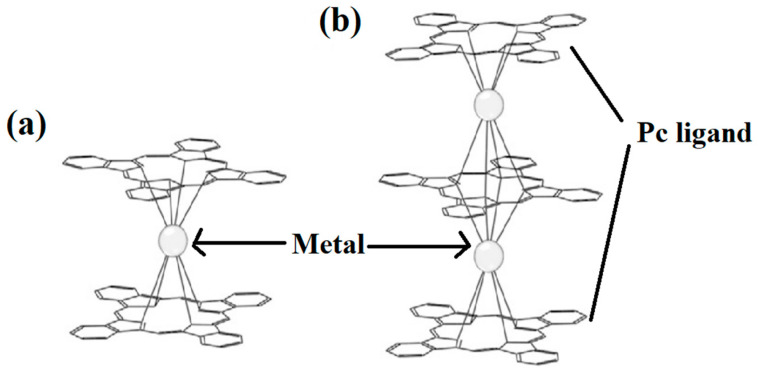
Structures of (**a**) double- decker metal Pcs, consisting of a metal ion sandwiched between two Pc ligands and (**b**) triple-decker metal Pcs, metal ions are stacked between sandwich-type Pc oligomers [30].

Pc is an organic semiconductor with 18π electrons, and it has been demonstrated that Ln-Pc double- and triple-decker complexes are capable of forming [18,19,20,21,22,23,24,25,26,27,28,29]. The structures are shown in Figure 2 [30].

Compared to conventional magnetic particles composed of metals, metal alloys, or metal oxides at the nanoscale, SMMs have many important advantages: (1) SMMs consist of relatively independent molecular units, so they have a single size and a fixed structure [31]. (2) SMMs are generally soluble in organic solvents, which makes it possible to obtain magnetic materials that were previously available only under special conditions in chemical solutions under ordinary conditions. (3) The magnetic characteristics of SMMs can be refined through metal ions and Pcs and by improving the synthesis methods [32].

SMMs generally consist of an intrinsic metal nucleus surrounded by an organic ligand shell. Lanthanide elemental metal ions with a high spin ground state are good choices for the preparation of molecular materials with SMMs. However, designing the SMMs of such ions requires care to optimize the spatial distribution of ligand electrons with respect to the ion.

### 2.2. Double-Decker Pc of Tb/Dy

**Figure 3:**
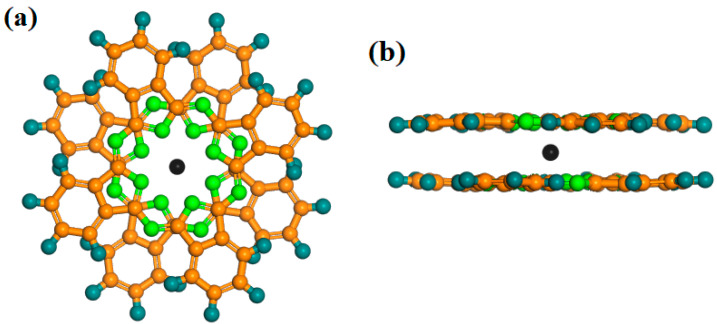
Diagrammatic sketch of [LnPc_2_]^−^ (Ln = Tb, Dy), the angle between Pc ligands is 45°. (**a**) Top view; (**b**) side view. Colors: (Ln = Tb, Dy), black; N, green; C, orange; H, navy blue.

To date, more than one hundred Ln-SMMs have been discovered and studied. Owing to their excellent physical properties, Ln-Pcs (Ln = Tb, Dy) are widely favored by researchers [33,34,35,36,37]. The model of Ln-Pcs is shown in Figure 3a,b, the Ln^3+^ (Ln = Tb, Dy) ion is located in the center of the molecule with two parallel Pc rings to form a sandwich structure molecule. The double-decker Ln-Pcs (Ln = Tb, Dy) has D_4d_ symmetry [33,34,38]. DyPc_2_ is similar in properties to TbPc_2_, which possesses an anisotropic U_eff_ of 410 cm^−1^ and a spin-orbit coupling quantum number of J = 6 [39].

Rare earth Pcs were discovered by Kirin and Moskalev. Notably, double-decker Ln-Pcs could also be achieved at that time [40,41]. The crystal structure data of LnPc_2_ (Ln = Tb, Dy) are shown in Table 1 [16]. TbPc_2_ belongs to the same P2_1_2_1_2_1_ space group as DyPc_2_, and the crystal parameters are close in size.

Dy ion-containing materials (such as magnetic resonance imaging, magnetostriction, and SMMs) have a wide range of promising applications in the magnetic field [42,43,44,45]. In SMMs, magnetic exchange interactions are important factors affecting the performance of SMMs, and early studies have shown that even very weak intermolecular magnetic exchange interactions can effectively suppress quantum tunneling effects and enhance the performance of SMMs [46,47]. Although the 4*f* electrons of rare earth ions are subject to the shielding effect of the outer electrons and the magnetic exchange between metal ions is relatively weak, this effect still has a significant impact on the properties of their SMMs.

**Figure 4:**
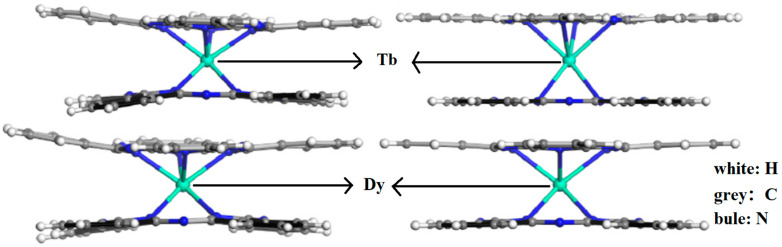
The structures of LnPc_2_ (Ln = Tb, Dy) compounds from X-ray diffraction (XRD) measurement (**left**) and density functional theory (DFT) calculation (**right**). In the DFT calculation, the PBE GGA correlation functional by Perdew-Burke-Ernzerhof (PBE) was the functional of choice, complemented by the empirical dispersion correction developed by Grimme [48].

Martínez-Flores et al. studied the geometries and electronic properties of LnPc_2_, as shown in Figure 4. They reported that unpaired electrons are transferred to Pc ligands [48], and the strong π-π interaction between intramolecular Pc rings becomes important for organic field effect transistors as intrinsic semiconductors compared to their monolayer analogs.

The magnetic coupling of TbPc_2_ molecules was reported by Corradini and coworkers. They placed TbPc_2_, single-layer graphene, and an Au single-layer on top of a Ni(111) magnetic substrate. They found that the superexchange coupling leads to a change in the antiferromagnetic signal [49].

### 2.3. Multi-Decker Pc of Tb/Dy

Ln_2_-SMMs are SMMs containing two lanthanide element ions forming a large collection, and double-nuclear Pcs SMMs containing Dy and Tb are more common. The radially contracted nature of the 4*f* orbitals of rare earth ions tends to lead to extraordinarily weak intramolecular exchange coupling in multinuclear lanthanide complexes. Therefore, for most multinuclear Ln_2_-SMMs, the magnetic origin is mainly a single-ion effect.

There is another class of double nuclear Ln_2_-SMMs that are trilayer structured Pc SMMs, and the chemical general formula of these molecules is [PcLn(μ-Pc_2_)Ln(Pc_3_)] when the ligand Pc can be heterocyclic. The spacing between the Ln ions in the molecule is approximately 0.36 nm, which makes it possible to study the effect of intramolecular *f-f* interactions on the dynamic magnetic properties, and the lanthanide ions have a significant role in the physical properties of triple-decker Pc compounds [50].

**Figure 5:**
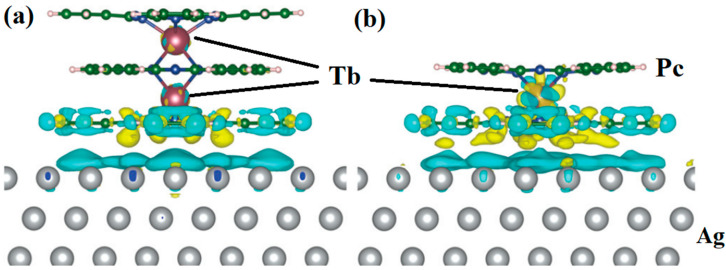
Calculated charge transfer between (**a**) Tb_2_Pc_3_ and (**b**) TbPc_2_ molecules and the Ag(111) surface obtained from DFT calculations. It used exchange correlation functional PBE + U with U = 5 eV for *f*-electrons of Tb and van der Waals interaction was approximated by the Tkatchenko-Scheffler dispersion correction method. The yellow and blue colors represent the accumulation and loss of density, respectively. The presence of blue density on the upper surface layer indicates substantial charge transfer from the metallic surface toward the molecule [34].

Hellerstedt et al. reported a method to form Tb_2_Pc_3_ from TbPc_2_. The structures are shown in Figure 5a,b. The different colors (yellow and blue) of the densities represent the charge redistribution. The formation of Tb_2_Pc_3_ provides a novel way to investigate and control magnetic interactions [34].

Ln_3_-SMMs can be divided into two main categories according to the structural arrangement of the metal ions: triangular and chain-like metal ion arrangements. Multinuclear Ln-SMMs are relatively rare in most rare earth elements because they are not easily synthesized due to their high nucleus numbers. Of course, Dy is the exception; the vast majority of rare earth SMMs with high nucleation numbers contain Dy, and the number of nuclei in Dy-SMMs can be as high as 50. However, in general, ligands for multinuclear Dy systems are not limited to Pcs.

This section focuses on the double-decker Pc of Tb/Dy and the multi-decker Pc of Tb/Dy, including its structure and the work of its predecessors. As expected, that was previously made, the discovery that Ln-SMMs can exhibit slow relaxation of the magnetization has initiated intensive interest in the SMMs containing lanthanide metals (4*f*). Herein, the Dy/Tb ion seems to be especially useful in this respect. Dy/Tb-radical family was considered and used over the last years as a bench for understanding the magnetism of the lanthanide ions and has given rise to many groundbreaking results in SMMs in recent years.

As will be further discussed in the next section, the Dy/Tb-Pcs are more common in the single-nuclear and bi-nuclear form, however, it is still necessary to study multi-nuclear Dy/Tb-SMMs.

## 3. Other Single-Molecule Magnets

**Figure 6:**
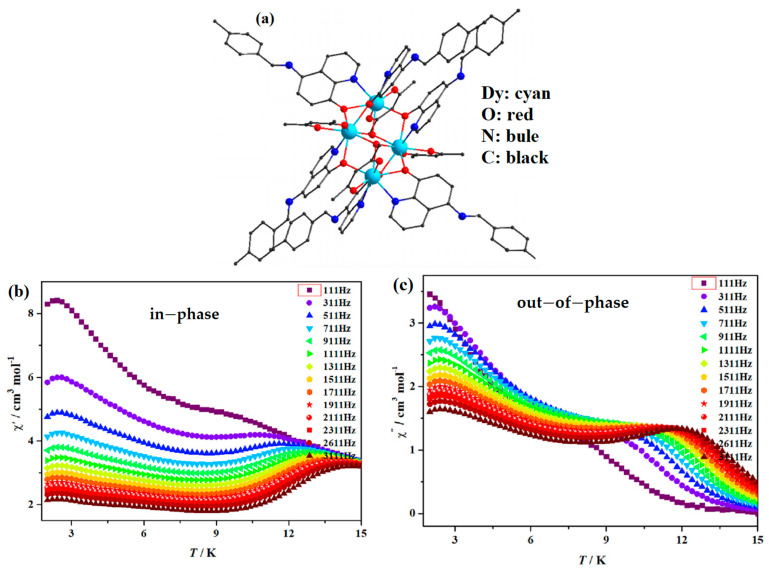
The schematic image: (**a**) Model of [Dy_4_(acac)_4_(*μ*_2_–L)_6_(*μ*_3_–OH)_2_]·2C_2_H_5_OH. Temperature dependence of (**b**) in–phase and (**c**) out–of–phase magnetic induction strength [51].

In 2020, Wang et al. reported the synthesis of [Ln_4_(acac)_4_(*μ*_2_-L)_6_(*μ*_3_-OH)_2_]·2C_2_H_5_OH (Ln = Tb and Dy). Its structure is shown in Figure 6a. They found that significant slow magnetic relaxation behavior occurred for [Dy_4_(acac)_4_(*μ*_2_-L)_6_(*μ*_3_-OH)_2_]·2C_2_H_5_OH with an anisotropic barrier of 82.1 K, as shown in Figure 6b,c [51]. For [Dy_4_(acac)_4_(*μ*_2_-L)_6_(*μ*_3_-OH)_2_]·2C_2_H_5_OH, below 15 K, both in-phase and out-of-phase become frequency dependent, and two distinct peaks for the out-of-phase ac signals are evident during the frequency range 311–3111 Hz, indicating the possible multiple relaxation processes existing in it.

In 2021, Wang et al. synthesized the {Dy_4_(acac)_4_L_4_} compounds. Through studying the relationship between ln (*τ*) and T^−1^, and combining the Arrhenius law ln(*τ*) = ln(*τ*_0_) + (∆E_eff_ /k_B_)T^−1^, they obtained that U_eff_ reaches 34.1 cm^−1^ and the preexponential factor reaches 6.92 × 10^−6^ s [52].

**Figure 7:**
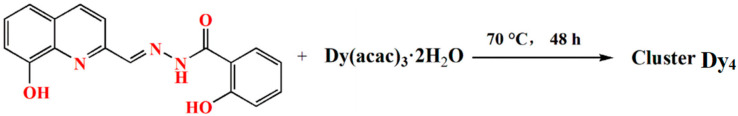
The synthesis steps of the Dy_4_ cluster [53].

A new Dy_4_ cluster based on a polydentate Schiff base ligand was reported by Shi et al. [53]. They found that the Dy_4_ cluster has obvious SMM behavior. Figure 7 displays the synthesis steps of the Dy_4_ cluster: Dy(acac)_3_·2H_2_O, H_3_L, CH_3_OH, CH_3_CN and CH_2_Cl_2_ were enclosed in a glass vial and heated to 70 °C for about 48 h. Then it was dropped to room temperature at a rate of about 5 °C/h, and the crystals Dy_4_ cluster was obtained.

When the number of metal ions in rare earth SMMs becomes more numerous, a variety of structural forms are produced, such as one-dimensional linear, sawtooth or polyhedral shapes. In addition, their magnetic properties can vary widely depending on various factors. The synthesis of multinuclear rare earth SMMs and research on magneto-structural relationships are interesting. Due to the large number of metal ions inside multinuclear rare earth SMMs, the complexity of the interactions between the metal ions increases exponentially compared to that of binuclear ones, and the properties of the SMMs can be affected.

**Figure 8:**
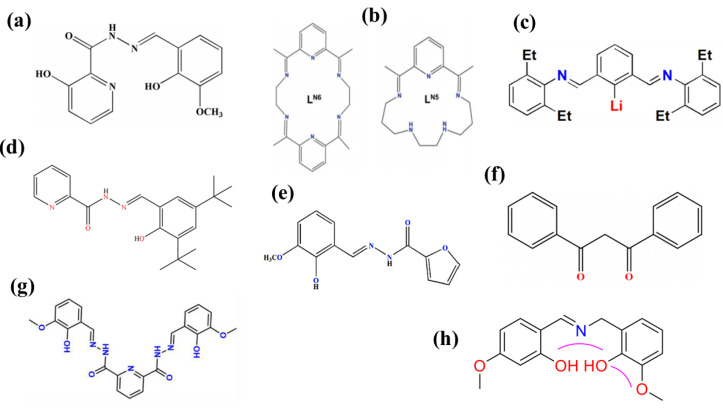
The structure of the Schiff base ligand (**a**) H_3_L [54]. (**b**) L^N5^ and L^N6^ [55]. (**c**) LiL^2^ [56]. (**d**) H_2_L [57]. (**e**) HL [58]. (**f**) Hdbm [59]. (**g**) H_4_Bmshp [60]. (**h**) H_2_hmp [61].

In addition to the widely studied Pc ligands, H_3_L [54], L^N5^ and L^N6^ [55], LiL^2^ [56], H_2_L [57], HL [58], Hdbm [59], H_4_Bmshp [60], H_2_hmp [61], are also hot spots of research. Schematic diagrams of their structure are shown in Figure 8.

Blagg et al. [62] reported a case of isopropanol-bridged Dy_5_-SMMs: [Dy_5_(μ_5_-O)(μ_3_-OiPr)_4_(μ-OiPr)_4_(OiPr)_5_], in which all the Dy^3+^ in the complex are hexa-coordinated and five Dy^3+^ form a positive tetragonal cone. The results show that the complex has the properties of SMMs below 50 K, and the flip U_eff_ is as high as 367 cm^−1^, which sets a new record for the flip U_eff_ of multinuclear SMMs at that time. Subsequently, Blagg et al. [63] reported another example of a tetranuclear rare earth SMM [Dy_4_K_2_O(Obi)_12_] and discovered a relaxation process involving the second excited state. [Dy_4_K_2_] forms an octahedron with two K ions in the cis position, and Dy^3+^ is six-coordinated, showing a deformed octahedral structure. It is found that [Dy_4_K_2_] has a two-step slow relaxation behavior by AC magnetization with U_eff_ values of 692 cm^−1^ and 316 K, respectively, and hysteresis lines can be observed below 5 K. Langley et al. [64] reported the first 4*d*-4*f* multinuclear SMMs [Ru_2_Dy_2_(OMe)_2_(O_2_CPh)_4_(mdea)_2_(NO_3_)_2_], and its U_eff_ was 10.7 cm^−1^.

**Figure 9:**
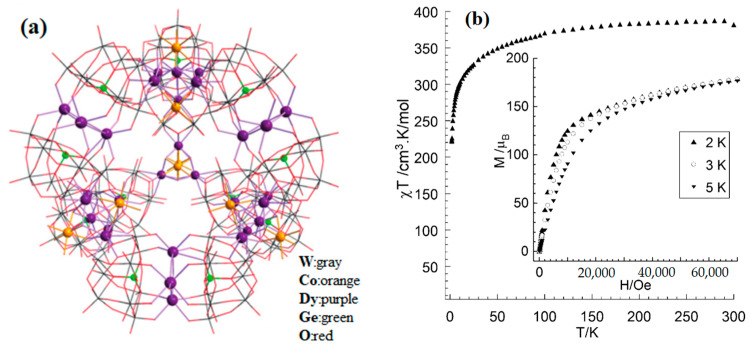
The schematic image: (**a**) Model of [Dy_30_Co_8_Ge_12_W_108_O_408_(OH)_42_(OH_2_)_30_]^56−^. (**b**) The curve of χT versus T. Inset: Curve of M versus H [65].

The SMM with the largest number of nuclei is the polymetallic oxonate [Dy_30_Co_8_Ge_12_W_108_O_408_(OH)_42_(OH_2_)_30_]^56−^ reported by the Powell group [65], which is self-assembled by six {W_9_Dy_3_W_9_} linkers and four {Co_2_Dy_3_} nodes with a very beautiful topology (Figure 9a). The magnetic susceptibility of polyanion [Dy_30_Co_8_Ge_12_W_108_O_408_(OH)_42_(OH_2_)_30_]^56−^ was investigated. Figure 9b shows the relationship between χT and T. As the temperature increases, χT rapidly increases in the early stage and slowly increases in the later stage. The curve trend of the field dependence of magnetization is indicative of significant anisotropy of [Dy_30_Co_8_Ge_12_W_108_O_408_(OH)_42_(OH_2_)_30_]^56−^ (Figure 9b, inset).

On the one hand, the regulation of Ln-SMMs helps to explore the relationship between structure and magnetism, which leads to a deeper understanding of the slow magnetic chirality mechanism of SMMs; on the other hand, the theory guides the experiment and the theory as a means to guide us to research compounds with better performance. For example, Long et al. gave qualitatively the intrinsic relationship between the rare-earth ion energy levels and the surrounding ligand field by studying the characteristics of the 4*f* charge density distribution corresponding to the ground state and different excited states of rare-earth single ions [66] to determine the ground state of the lanthanide ions with high magnetic anisotropy. Later, several researchers proposed and refined the use of electrostatic field models to predict the quantum axis of rare earth ions [67,68]. These works provide important theoretical guidance for the design of Ln-SMMs. In addition, in order to give an insight into especially ‘experimentally difficult’ systems recourse to theoretical tools is a very common and fruitful approach [48].

### 3.1. Acetylacetone-Based SMMs

Acetylacetonate ligands belong to the 1,3-dicarbonyl group, and there are keto-enol interchangeable structures within the molecule. Therefore, there are functional groups such as hydroxyl and carbonyl groups within the ligand as well as active H atoms at the α-position of unsaturated double bonds. In particular, the ligand removes an H atom to form a stable bidentate chelate structure in an alkaline environment, which can form stable complexes with metal ions. It is characterized by a simple synthesis route, high yield and strong stability.

**Figure 10:**
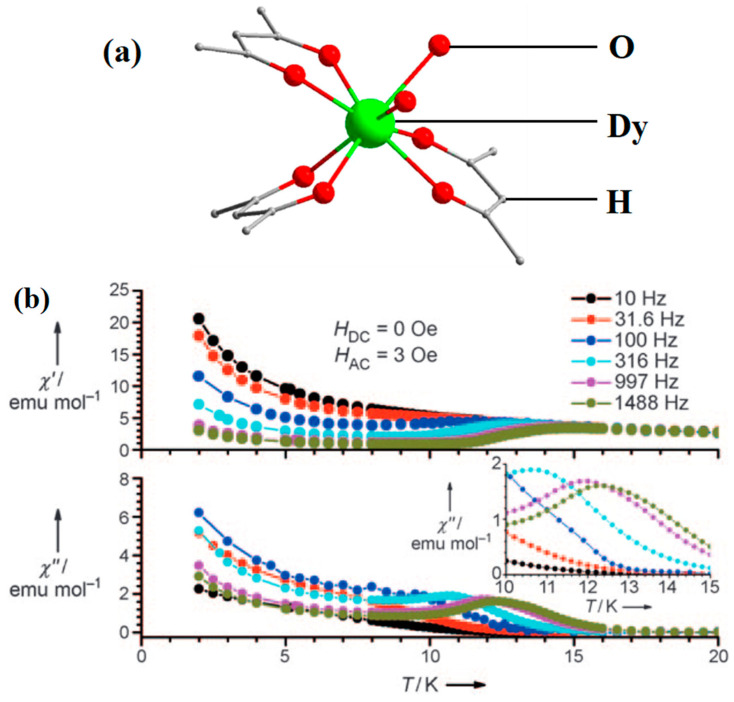
The schematic image: (**a**) Model of [Dy(acac)_3_(H_2_O)_2_]. (**b**) The relationship curves of temperature and ac susceptibility at frequencies from 10 to 1488 Hz for the undiluted Dy compound. Dy green, H atoms and solvent molecules or ligands are omitted [69].

Jiang et al. [69] synthesized an example of a neutral mononuclear complex [Dy(acac)_3_(H_2_O)_2_] using acetylacetone and determined its magnetic properties, which kicked off the study of acetylacetone-based SMMs. As shown in Figure 10, the compound has a local symmetry close to D_4d_, and the eight coordinated O atoms form a deformed tetragonal anti-prismatic coordination configuration, in which the magnetic anisotropy of the Dy^3+^ is enhanced under the crystal field.

Gao et al. [70] reported Dy-(1,1,1,2,2,3,3-heptafluoro-7,7-dimethyl-4,6-octadione) complexes, named Dyfod_3_bpy (Dy_1_). Dy_1_ melts at 90 °C and evaporates at 269 °C to form Dyfod_3_phen (Dy_2_). They found that Dy_2_ maintains the SMM properties, but the relaxation barrier shifts from 87 K to 122 K.

### 3.2. Polyacid-Based SMMs

Polymetallic oxides (referred to as polyacids) are a class of inorganic oxygen-containing acids that can be used to assemble novel transition metal or rare earth metal complexes. Polymetallic acids have oxygen-rich surfaces and high negative charges, and their absent sites can provide a suitable coordination environment for rare earth ions. Polyacids usually consist of antimagnetic vanadium, molybdenum, tungsten and niobium ions in the highest oxidation state. Therefore, these nanosized polyacids can be considered ideal antimagnetic shells for separating magnetic spin carrier components and can dilute magnetic units and effectively shield magnetic exchange between spin carriers.

**Figure 11:**
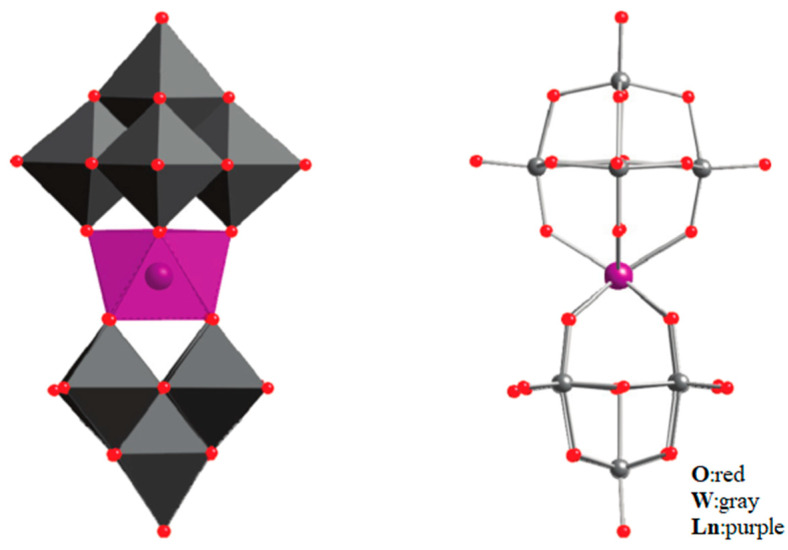
The schematic image:model of [Ln(W_5_O_18_)_2_]^9−^. The left is the polyhedral type, and the right is the ball-and-stick type [71].

**Figure 12:**
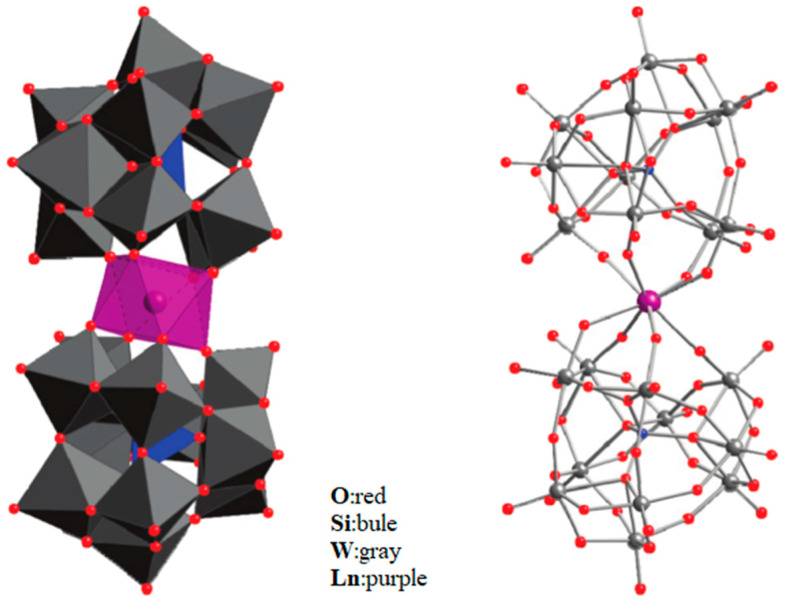
The schematic image: model of [Ln(β_2_-SiW_11_O_39_)_2_]^13−^. The left is the polyhedral type, and the right is the ball-and-stick type [72].

AlDamen et al. reported a series of lanthanide polyoxometalate compounds: [LnW_10_O_36_]^9−^ (Ln = Tb, Dy, Ho, Er) (Figure 11) [71] and [Ln(β_2_-SiW_11_O_39_)_2_]^13−^ (Ln^III^ = Tb, Dy, Ho, Er, Tm, and Yb) (Figure 12) [72]. Interestingly, [ErW_10_O_36_]^9−^ is the first example of an Er-based heteropolyacid SMM. Ln can be seen to be in a ligand field with approximate D_4d_ symmetry but exhibits a completely different behavior from [LnPc_2_]^−^ as an SMM.

**Figure 13:**
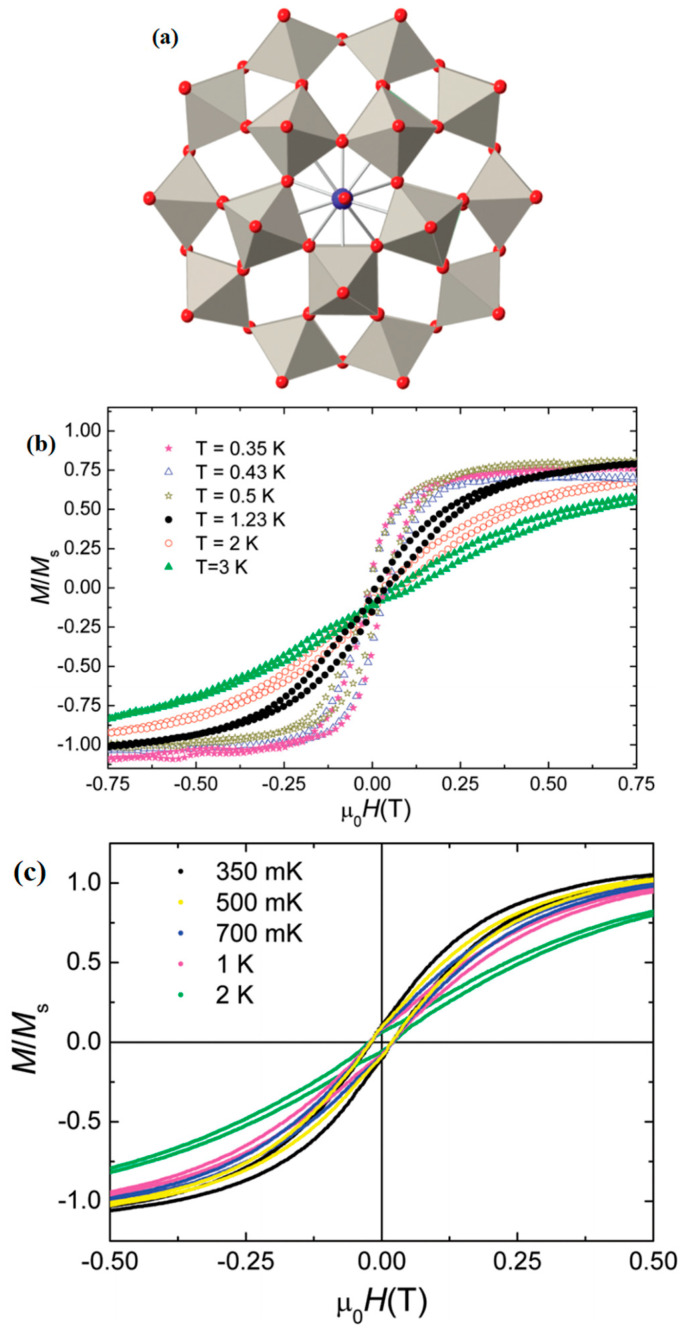
The schematic image: (**a**) Model of [LnP_5_W_30_O_110_]^12−^. (**b**) Hysteresis curves for DyW_30_. (**c**) Magnetization hysteresis loops of HoW_30_ [73].

Cardona-Serra et al. [73] reported [LnP_5_W_30_O_110_]^12−^ (Ln = Dy, Ho) with 5-fold symmetry. When Ln = Dy, Ho, it shows magnetic hysteresis at low temperatures and obviously big off-diagonal anisotropy parameters $A_{6}^{5}$. its structure is shown in Figure 13.

### 3.3. SMMs in the Pentagonal Biconical Configuration

Both the different lanthanide centers and different ligand fields can significantly influence the magnetic anisotropy of SMMs [74]. The most common central metal ion in multinuclear monomolecular magnets is Dy. In recent years, several cases of monomolecular magnets with pentagonal bipyramidal (PB) structures as confirmed by other researchers or groups, all showing crystal fields with high axial symmetry of D_5h_ [75,76,77,78]. In 2016, an example of an SMM [Dy(O^t^Bu)_2_(py)_5_][BPh_4_] [79] with a perfect pentagonal bipyramidal configuration was reported by Zheng et al., whose U_eff_ can reach 1269.3 cm^−1^ and T_B_ of 14 K.

**Figure 14:**
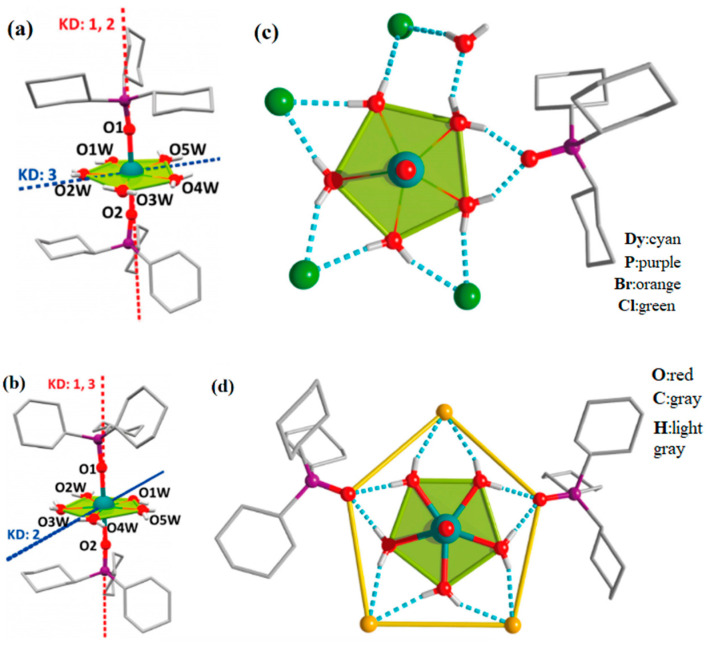
Crystal structures of (**a**) [Dy(Cy_3_PO)_2_(H_2_O)_5_]Cl_3_·(Cy_3_PO)·H_2_O·EtOH. (**b**) [Dy(Cy_3_PO)_2_ (H_2_O)_5_]Br_3_·2(Cy_3_PO)·2H_2_O·2EtOH (Cy_3_PO = tricyclohexylphosphine oxide). Coordination environment (**c**) Corresponding to (**a**). (**d**) Corresponding to (**b**). H atoms of the ligands are omitted for clarity. Red and blue dashed lines are the main anisotropy axes in the ground Kramers doublet and the excited Kramers doublet, respectively [80].

As shown in Figure 14, Chen et al. [80] took advantage of the local symmetry of D_5h_ to increase the magnetic T_B_ of Dy single ion magnets to 20 K for the first time. They synthesized the U_eff_ of [Dy(Cy_3_PO)_2_(H_2_O)_5_]Cl_3_·(Cy_3_PO)·H_2_O·EtOH is 472 cm^−1^ and [Dy(Cy_3_PO)_2_ (H_2_O)_5_]Br_3_·2(Cy_3_PO)·2H_2_O·2EtOH (Cy_3_PO = tricyclohexylphosphine oxide) at 543 cm^−1^.

The pentagonal biconical symmetric configuration of Dy SMMs has a high axial magnetic anisotropy, which gives it the potential to obtain higher U_eff_. In 2020, Canaj et al. [55] reported [Dy^III^(L^N5^)(Ph_3_SiO)_2_](BPh_4_)·CH_2_Cl_2_, which has an anisotropy barrier of 1108 cm^−1^. Moreover, when five N atoms are replaced in the equatorial plane, U_eff_ increases.

Yuan et al. reported [Dy_4_L_4_(Ph_2_acac)_2_(OH)_2_(DMF)_2_] (H_2_L = (E)-2-(((2-hydroxyphenyl)imino)methyl)-6-methoxyphenol; Ph_2_acacH = β-diketones dibenzoylmethane) and [Dy_4_L_4_(acac)_2_(OH)_2_(DMF)_2_]⋅2CH_3_CN (acacH = acetylacetone). They derived that both of them have SMM behavior. Their U_eff_ values are 50.1 cm^−1^ and 147.2 cm^−1^, respectively [81]. In view of the outstanding performance for these Dy/Tb SMMs, in the last several years lots of new structures have been reported. In this section, we introduced and summarized: Acetylacetone-based SMMs, Polyacid-based SMMs, and SMMs in the pentagonal biconical configuration. Each type of SMMs has its own unique structure and properties. All these results lately propitiate a new era for SMMs and can provide some research ideas for studying other ligand types of SMMs. In addition, we have summarized some basic properties of common SMMs for the convenience of readers, as shown in Table 2.

## 4. Preparation Technology

The main methods for the study of Ln-Pcs are as follows: (1) Halogen synthesis method; (2) Metallation of free-base ligands; (3) Mono Pc-based techniques; and (4) Axial substitution at the metal center [82,83]. In 1965, Kirin and Moskalev studied the reaction of rare earth acetates and phthalonitrile (PN) at 280–290 °C [40,41,84]. This led to the formation of Ln-Pcs (Ln = Pr, Nd, Er, Lu) [85,86,87].

**Figure 15:**
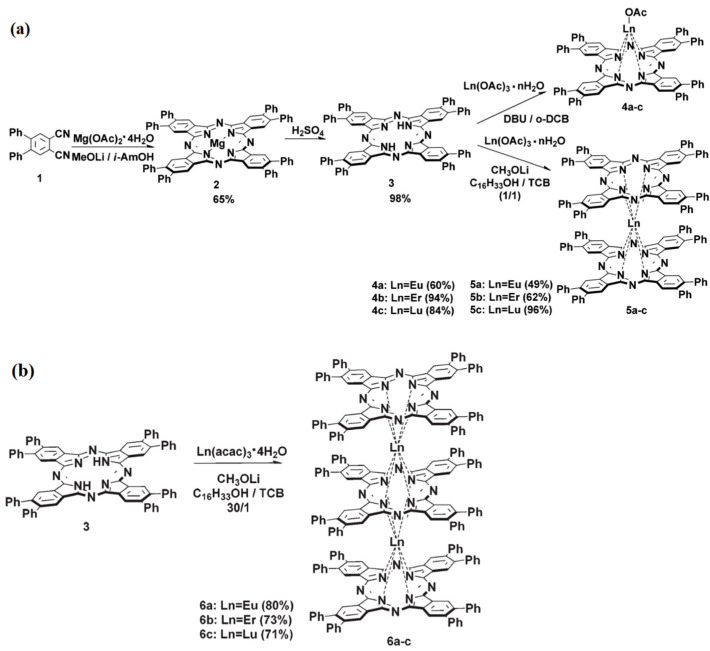
Steps for preparing Pcs. (**a**) Single- and double-decker. (**b**) Triple-decker [83].

Dubinina et al. proposed a method for preparing Ln-Pcs in an alcohol/C_12_H_8_Cl_4_N_2_ (TCB) mixture. In this method, the Ln-Pcs were synthesized by a three-step procedure (Figure 15a). Then, phenyl-substituted Pc complexes 6a-c were synthesized selectively from ligand 3 and acetylacetonate salts of the corresponding lanthanides in a mixture of cetyl alcohol-TCB (Figure 15b). It can prepare Ln-Pcs of phenyl [83].

Simple heating of the single-decker lutetium complex to 400 °C in a vacuum (1 Torr) produces a triple-decker complex. Another method is sublimation under high vacuum (10^−6^ Torr) at 300–420 °C of the reaction obtained from template condensation between PN and a series of rare earth acetates (Ln = La, Nd, Eu, Gd, Dy, Er, Yb, and Lu) [88].

The development of single-molecule materials is accompanied by the development of material preparation technology. Methods such as vacuum evaporation, spin coating and the Langmuir-Blodgett technique have been widely used to study thin films of MPcs. For example, LnPc_2_ (Ln = Tb and Y) was synthesized using a solvothermal method [89].

**Figure 16:**
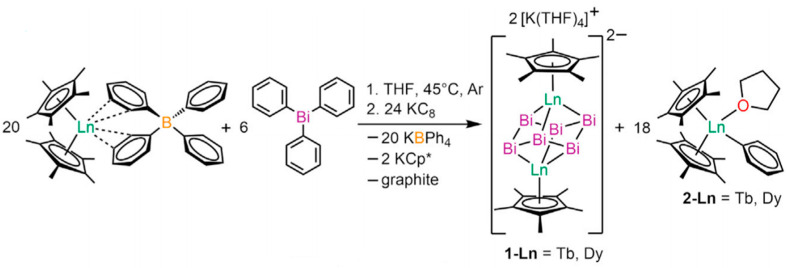
The schematic image: Synthetic scheme for [K(THF)_4_]_2_[Cp*_2_Ln_2_Bi_6_] (Ln = Tb, Dy) [90].

In 2022, Zhang et al. synthesized two bismuth-cluster-bridged lanthanide compounds, [K(THF)_4_]_2_[Cp*_2_Ln_2_Bi_6_] (Cp* = pentamethylcyclopentadienyl; 1-Ln, Ln = Tb, Dy), through the solution organometallic method (Figure 16). The relationship between *τ* and T^−1^ of [K(THF)_4_]_2_[Cp*_2_Ln_2_Bi_6_] was studied. They found that the lanthanide centers form strong ferromagnetic interactions between lanthanides, which lead to magnetic blocking and open hysteresis loops for super exchange-coupled SMMs comprising solely lanthanide ions [90].

The discovery of the magnetic properties of Pc rare earth-like compounds has stimulated research in this field and is still one of the hot spots for the exploration of new magnetic materials [91,92]. The commonly used magnetic SMMs test system is the magnet property measurement system (MPMS), which is composed of a detection system, software operating system, temperature control system, magnetic control system, sample operating system and gas control system. MPMS can perform tests such as Direct Current magnetization, Alternating Current magnetization and low temperature hysteresis lines [93].

To end this section, we would like to emphasize that the development of preparation technology is of great significance for the study of SMMs. This section summarized and introduced the main methods for the study of Ln-Pcs as follows: (1) Halogen synthesis method; (2) Metallation of free-base ligands; (3) Mono Pc-based techniques; and (4) Axial substitution at the metal center, these synthesis methods are more common and practical. The research on SMMs with new performance needs to be synthesized and validated through experimental preparation techniques, and progress towards the goal of practical quantitative production.

## 5. Performance of Single-Molecule Magnets

### 5.1. Magnetic Origin

SMMs constructed with rare earth metal ions have relatively large magnetic moments and magnetic anisotropy due to their special electron layer structure: *f* electrons have large unquenched orbital angular momentum.

**Figure 17:**
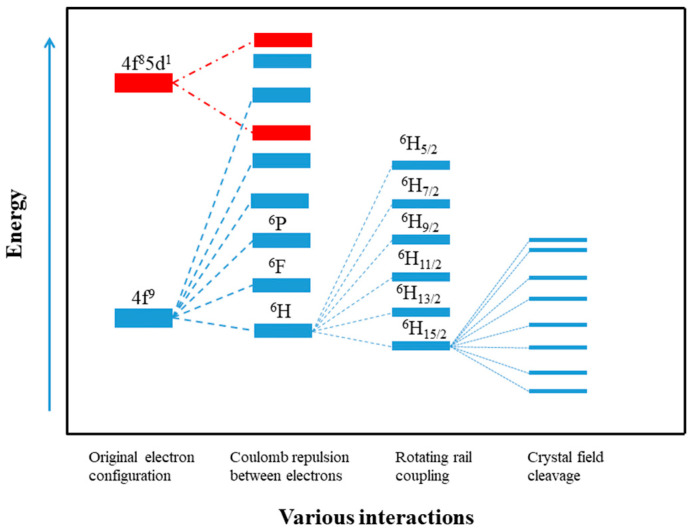
Effect of different interactions on energy level splitting of the degenerate 4*f*^9^ configuration of free Dy ions (from left to right, interaction is from strong to weak) [94].

To understand the magnetic origin and analyze the key factors influencing the magnetic behavior, the microscopic relaxation can be analyzed by a macroscopic model (Figure 17). The origin of the single-molecule magnetic behavior in lanthanide compounds is more sophisticated due to the spin-orbit coupling of the lanthanide metal ions that generates angular momentum J. Because the spin-orbit coupling energy is usually greater than the effect of the crystal field for 4*f* lanthanides and actinides, it is important to consider the spin-orbit coupling quantum number J. For the trivalent dysprosium ion with a 4*f*^9^ electron configuration, its free ion produces the ground state term ^6^H under the Coulomb repulsion between electrons, containing 66 energy levels, at which point they can all be considered to be simplicial. The presence of the spin-orbit coupling leads to further cleavage of this term, producing a series of nonsimple branches, the lowest energy of which is ^6^H_15/2_; if it is placed in a crystal field of certain symmetry, the crystal field action drives the spin ground state J = 15/2 branch to continue cleavage into m_J_ = ±15/2, ±13/2, ±11/2... ±1/2 and so on for the energy levels [93]. From the point of view of electronic structure, Dy-SMMs have been widely studied in recent years because the trivalent dysprosium ion has a large spin value (s = 5/2) and a large orbital angular momentum (l = 5) combined with a total orbital angular momentum J = 15/2, which leads to a particularly large magnetic anisotropy. Figure 17 depicts the influence of various interactions on the energy level splitting of the free Dy^3+^ simplicial 4*f*^9^ group state [94].

Most rare earth metal ions still exhibit the single-ion nature of the rare earth metal ions themselves in systems of synthetic polymers or clusters because of the shielding effect of the f electrons in the outer *s* and p electrons, which makes the magnetic interactions relatively weak again. Although the magnetic interaction between rare earth metal ions is weak, it still makes a significant contribution to its relaxation mechanism.

**Figure 18:**
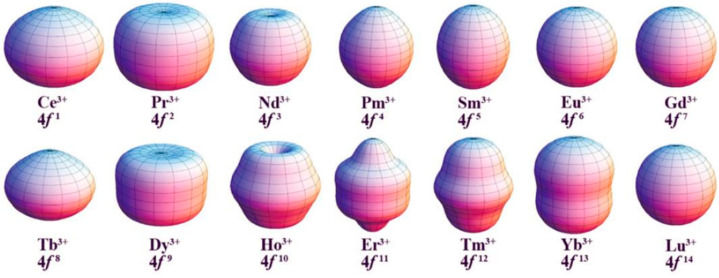
4*f* electron density distribution of corresponding Ln^III^ ions in their Ising limit state [95].

To clearly understand how to achieve maximum magnetic anisotropy for a specific lanthanide ion, Long’s group proposed a theoretical model based on the Ising limit state of various lanthanide ions through theoretical calculations (Figure 18) [66,95]. The model uses a quadrupole approximation calculation to describe the ground state charge density distribution corresponding to the eigenstates of various lanthanide ions. Due to the strong angular dependence of the 4*f* orbitals, the 4*f* electron charge density distribution of the lanthanide ions is not spherical but shows an anisotropic ellipsoidal shape [66]. By further drawing on the electrostatic model of effective point charges, it can be visualized that some of the lanthanide ion charge density distributions are flat and long (Pm^3+^, Sm^3+^, Eu^3+^, Er^3+^, Tm^3+^, Yb^3+^), some are flat (Ce^3+^, Pr^3+^, Nd^3+^, Tb^3+^, Dy^3+^, Ho^3+^) or are isotropic spheres (Gd^3+^) [95].

In the case of lanthanides with an oblate (squeezed along the axial direction) electron distribution, such as [Pc_2_Tb]^−^·TBA^+^ and [Pc_2_Dy]^−^·TBA^+^, the axial position of the ligand electrons is particularly favorable to produce considerable magnetic anisotropy. Notably, Dy-SMMs with different structural types will significantly influence the U_eff_ of SMMs. Such as, a centrosymmetric defect dicubane is found to show a remarkably large anisotropic barrier of 170 cm^−1^ [96].

**Figure 19:**
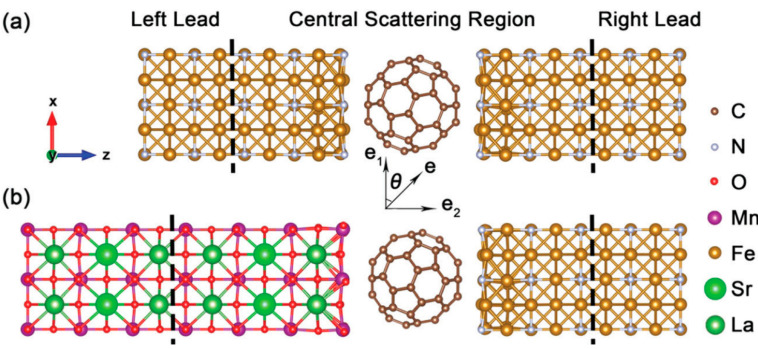
The models of (**a**) Fe_4_N/C_60_/Fe_4_N and (**b**) La_2/3_Sr_1/3_MnO_3_/C_60_/Fe_4_N [103].

The adsorption of Pcs can form spinterfaces by the interfacial coupling effect, which may change the magnetic properties of molecules and ferromagnetic substrates [97,98,99,100,101,102,103]. From Figure 19, we can see the Fe_4_N/C_60_/Fe_4_N and La_2/3_Sr_1/3_MnO_3_/C_60_/Fe_4_N models. Interestingly, the poles of La_2/3_Sr_1/3_MnO_3_/C_60_/Fe_4_N can be switched, which is useful for studying the function of spintronic devices [103].

Additionally, Tb ions and Dy ions have remarkable magnetic anisotropy in strongly axial ligand fields. This is because they have an oblate-shaped electron density [56,104]. The magnetic properties of Dy ions are more prominent than those of lanthanide ions, and using Dy-SMMs as a model, many novel results were found [7,105].

### 5.2. Magnetic Properties of SMMs

**Figure 20:**
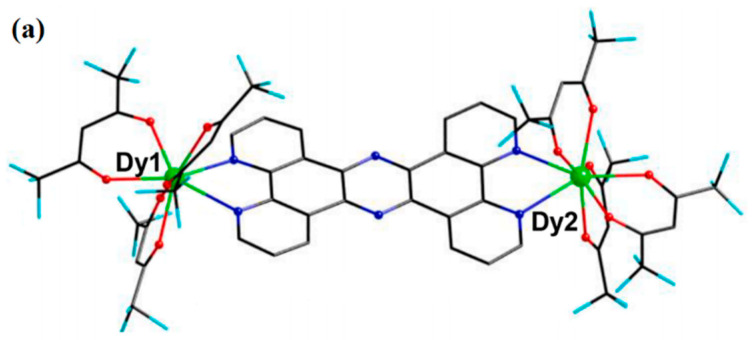

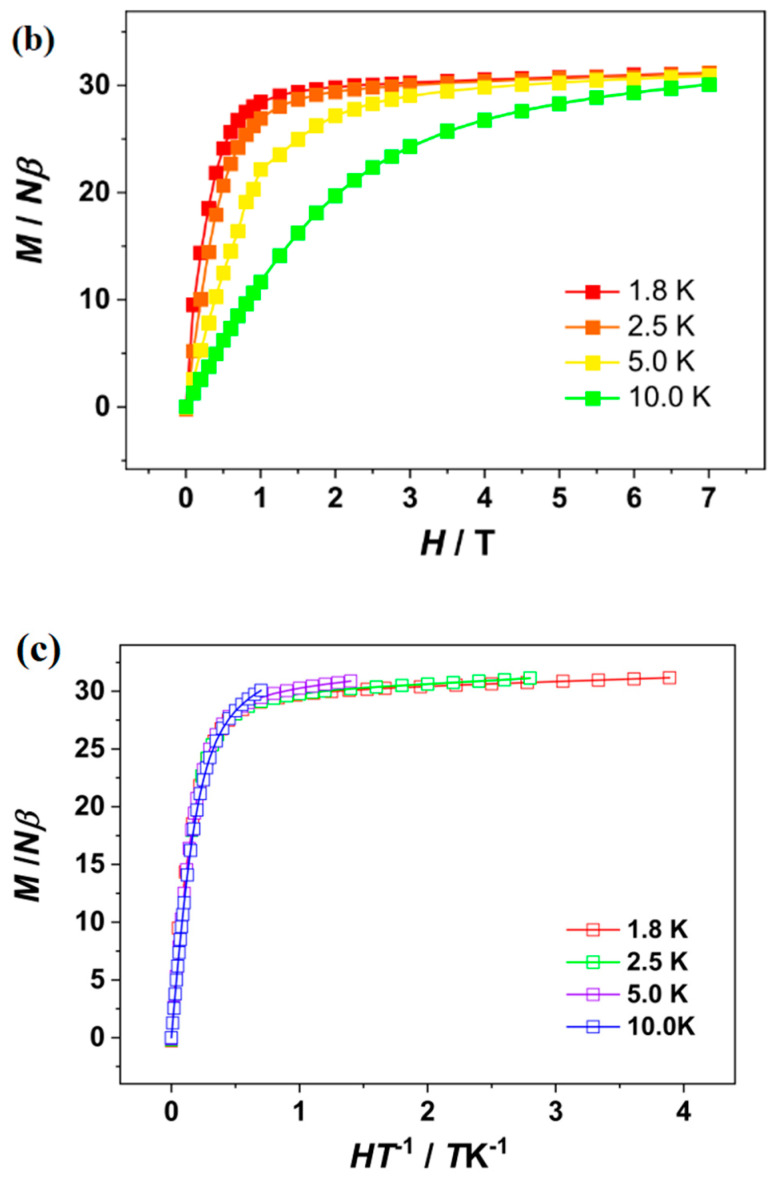
The schematic image: (**a**) Model of [Dy_2_(hfac)_6_(tpphz)]·CH_2_Cl. (**b**) Plots of M-H of [Dy_2_(hfac)_6_(tpphz)]·CH_2_Cl_2_. (**c**) Plots of *M-HT*^−1^ of [Dy_2_(hfac)_6_(tpphz)]·CH_2_Cl [106].

The magnetic properties of SMMs mainly include magnetic anisotropy, U_eff_, and T_B_. In 2023, Zhu et al. prepared a new compound [Dy_2_(hfac)_6_(tpphz)]·CH_2_Cl_2_, which has a big π-conjugated bridging ligand (Figure 20a). Magnetic measurements revealed that [Dy_2_(hfac)_6_(tpphz)]·CH_2_Cl_2_ possesses zero field SMM behavior with a single magnetic relaxation process. In Figure 20b, all curves rise rapidly before 1 T. As *H* increases, the curves rise slowly until the *H* value is 7 T. Furthermore, no superposition could be observed among the corresponding M-HT-1 curves (Figure 20c). All these results suggest significant magnetic anisotropy [106,107,108].

The current effective method to study the magnetic anisotropy of Dy-systems (triangular Dy_3_ and planar Dy_4_) is the Post-Hartree-Fock ab initio method. This method can not only get the energies of the multiplets but also determine the anisotropy axes and the *g* tensors for the lowest Kramers doublets of each dysprosium site [109,110].

**Figure 21:**
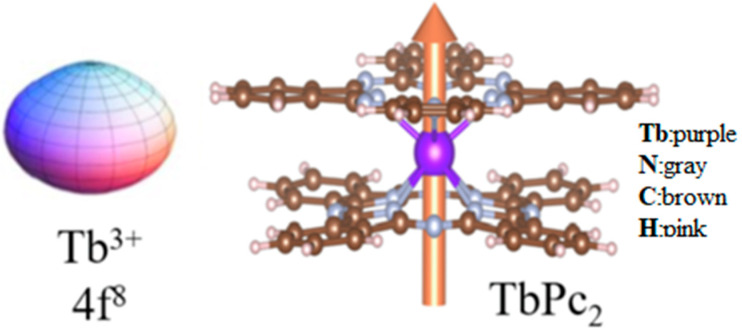
The model of Tb^3+^ ions in the Ising-limit state (**left**). The structure of TbPc_2_ (**right**). The golden arrow represents the magnetic anisotropy axis [111].

Ruan et al. analyzed the relationship between the magnetic properties and the TbPc_2_ film, as shown in Figure 21. The results show that when the growth temperature is 150 °C, the TbPc_2_ film has significant magnetic anisotropy; that is, the out-of-plane magnetic moments are obviously greater than the in-plane magnetic moments [111].

The more complex the molecular structure of SMMs, the less pronounced their properties become. This is because the more lanthanide ions there are in SMMs, the more difficult it is to control the coordination environment and the more complicated the situation.

Crystal field effects arise mainly from the Coulombic interactions of the central ion and ligand. The crystal field effect is closely related to Ln-SMMs because it is extremely influential for the splitting of multiple states. Furthermore, the “ion field” (4*f*-electron shell from the metal ion) and the “crystal field” (ligand shell from the ligand atom) constitute the coordination compounds.

Small changes in the coordination environment of rare earth ions will obviously influence the magnetic properties of the constructed complexes. Therefore, this characteristic of Ln-SMMs, which is extremely sensitive to the coordination environment, can be used to regulate the magnetic properties of Ln-SMMs. In essence, the regulation of Ln-SMMs includes two aspects: (1) the regulation of uniaxial magnetic anisotropy and (2) the regulation of intermolecular interactions. From the point of view of experimental design, the modulation methods that can be adopted are mainly (1) modulation of metal-centered Ln (Ln = Tb, Dy) ions and (2) modulation of the coordination environment (external field modulation).

In 2020, Chibotaru et al. used complete active space self-consistent field methods to study the magnetic anisotropy of the divalent lanthanide oxide LnO (Ln = Tb, Dy).

**Figure 22:**
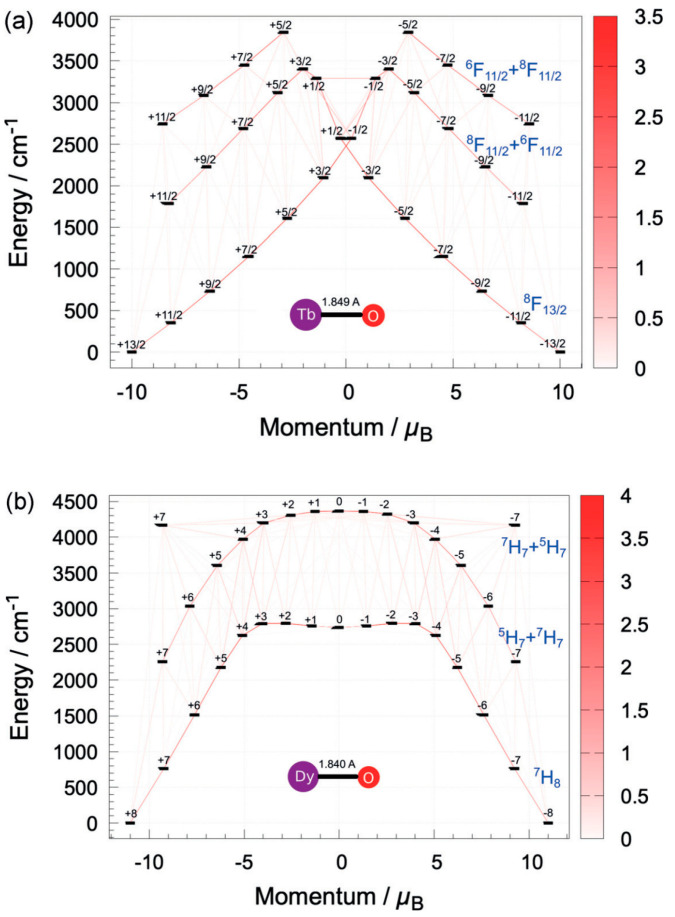
The relationship between the energy and momentum of (**a**) TbO and (**b**) DyO [112].

From Figure 22, we can see that the barrier of magnetization blocking sketches the contours of the relaxation pathway linking all doublet states arising from the ground atomic multiplet. This is similar to the case when the group of levels belonging to the ground atomic multiplet overlaps with the states from the excited atomic multiplet. The highest magnetic U_eff_ obtained by theoretical calculation exceeds 3000 cm^−1^, which qualitatively improves the expected performance of SMMs and provides a theoretical basis for the experimental synthesis of divalent Ln-SMMs [112].

It is worth noting that Ln-SMMs are an important model for studying the high anisotropic barriers of SMMs. The anisotropic barrier records are constantly being refreshed for Ln-SMMs, for example, defect-dicubane Dy_4_ (170 K) [113], linear Dy_4_ (173 K) [113] and pyramid Dy_5_ (528 K) [62].

**Figure 23:**
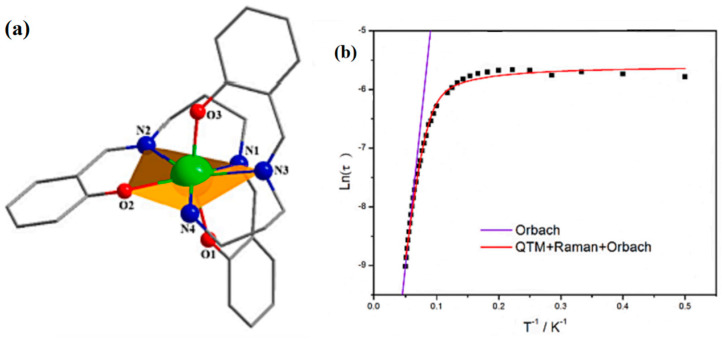
The schematic image: (**a**) The structure and (**b**) ln(*τ*) versus T^−1^ of [Dy(L_1_)(L_2_)] [114].

In 2023, Chen et al. reported [Dy(L_1_)(L_2_)] (HL_1_ = (E)-2-(((3-aminopropyl)imino)methyl) phenol, H_2_L_2_ = 2,2′-((1E,1′E)-(propane-1,3-diylbis(azaneylylidene))bis(methaneylylidene))diphenol) compounds (Figure 23a). Combined with Equation (1) and Figure 23b, the magnetic characterizations reveal that the material exhibits slow magnetic relaxation behavior with U_eff_ = 95.98 cm^−1^ [114].

Equation (1) expresses the relationship of ln(*τ*) versus T^−1^ for [Dy(L_1_)(L_2_)] [114]:

(1) $$\tau -1\mathrm{obs}=\tau \frac{-1}{QTM}+CT^{n}+\tau^{-1}0\exp\left(-U_{eff}/T\right)$$

When *n* is 1.8, *U_eff_* = 87.6 cm^−1^ and *τ*_0_ = 1.96 × 10^−6^ s for [Dy(L_1_)(L_2_)].

Due to the complex magnetic relaxation behavior of TbPc_2_ near the zero field at low temperatures and the quantum tunneling effect of magnetization, the remanence and coercivity of TbPc_2_ at low temperatures are also very small, showing a “butterfly” hysteresis line, which seriously hinders the application of TbPc_2_ SMMs for the preparation of high-density memory devices, such as the effect of exchange coupling between different substrates [39,115,116].

SMMs have magnetic bistability at the molecular level and exhibit slow magnetic relaxation over U_eff_ below the T_B_. When there is an external magnetic field, Zeeman splitting occurs, and the original simplex Population number will be destroyed. Therefore, the magnetization intensity vector sum will no longer be equal to zero, so SMMs have magnetic bistability. When the applied magnetic field is withdrawn, the magnetization intensity is reoriented, the process of reorientation then takes place to overcome U_eff_, and the temperature at which the magnetic moment is frozen is called the T_B_ [96].

In recent decades, researchers have been working on increasing U_eff_ and improving T_B_. The T_B_ increases from the previous 60 K [117] to 80 K [118]. Although this result is very encouraging, it is still far from room temperature (300 K), which becomes one of the biggest obstacles limiting the realization of SMMs for practical applications. The reason for the relatively high U_eff_ and T_B_ of the high-performing SMM system is mainly attributed to the significant magnetic anisotropy possessed by the molecular magnets, and the lanthanide ion magnetism comes from the magnetic anisotropy caused by the strong intrinsic spin-orbit coupling. Conventional magnetic materials are difficult to improve due to the limitation of the superparamagnetic effect, but the emergence of molecular-based magnets can solve this problem precisely. SMMs can be used to make ultrahigh-density storage materials owing to their tiny nanometer sizes and obvious magnetic behavior [119]. Modulation of the ligand field of Ln-SMMs allows the construction of high-performance SMMs. Most Ln-SMMs (Ln = Tb, Dy) have high U_eff_ [12,13,73].

**Figure 24:**
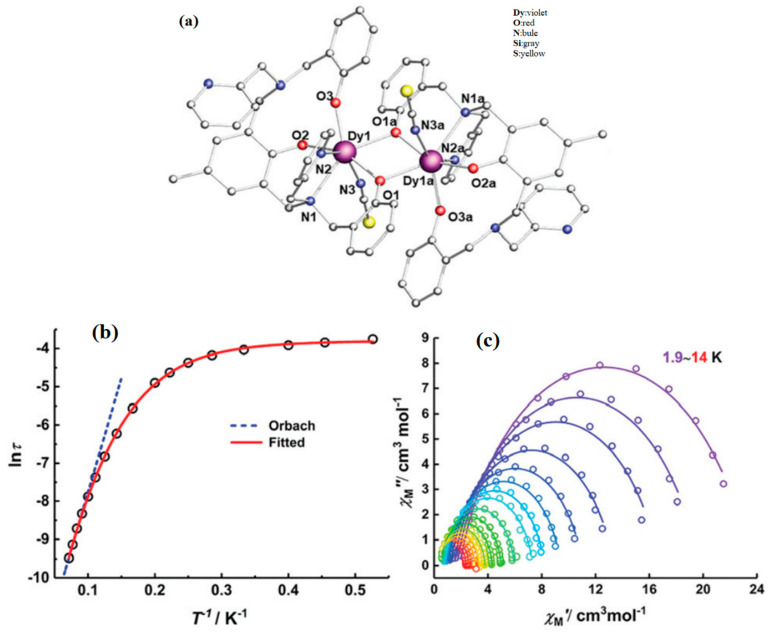
The schematic image: (**a**) Structures of [Dy_2_(HL)_2_(SCN)_2_]·2CH_3_CN. For brevity, H is omitted. (**b**) Arrhenius plots of relaxation time data. (**c**) Cole-Cole plots under a zero-dc field for [Dy_2_(HL)_2_(SCN)_2_]·2CH_3_CN. Solid lines correspond to the best fits [54].

Wu et al. assembled [Dy_2_(HL)_2_(SCN)_2_]·2CH_3_CN complexes using the H_3_L multidentate ligand (Figure 24a). According to the equation to fit the data: $\tau_{obs}{}^{-1}=\tau_{QTM}{}^{-1}+CT^{n}+\tau_{0}{}^{-1}\exp\left(-U_{eff}/T\right)$, where the Orbach parameters are *U_eff_* and *τ_0_*, the Raman parameters are *C* and *n*, and the rate of quantum tunneling of magnetization (QTM) is $\tau_{QTM}{}^{-1}$. The plot of ln (*τ*) versus 1/T exhibits a linear regime at high temperatures in Figure 24b, suggesting the dominance of the Orbach relaxation process. The quantum tunneling of magnetization (QTM) and Raman processes probably play the leading role at low temperatures, which is verified by the presence of curvature and temperature-independent regimes [54,120]. The Cole-Cole curves can be fitted using the generalized Debye model. The Dy centers in the complexes display capped octahedron coordination geometries and behave as an SMM, as shown in Figure 24c [54].

**Figure 25:**
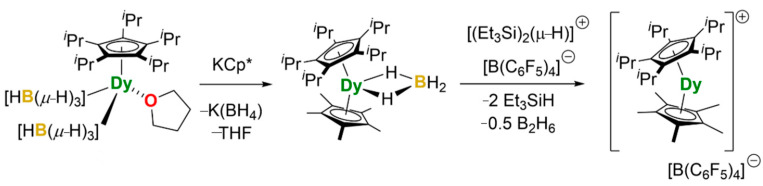
The schematic image: The synthesis method of [(Cp^iPr5^)Dy(Cp*)][B(C_6_F_5_)_4_] [118].

**Figure 26:**
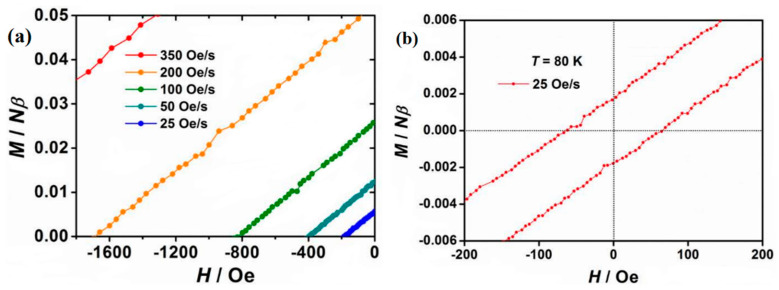
The schematic image: (**a**) Expansion of the hysteresis loops at 77 K of [(Cp^iPr5^)Dy(Cp*)][B(C_6_F_5_)_4_]. (**b**) Hysteresis loops at 80 K of [(Cp^iPr5^)Dy(Cp*)][B(C_6_F_5_)_4_] [118].

Guo et al. studied a compound [(Cp^iPr5^)Dy(Cp*)]^+^ (Cp^iPr5^ = penta-iso-propylcyclopentadienyl, Cp* = pentamethylcyclopentadienyl), as shown in Figure 25. The hysteresis temperature was increased to 80 K, reaching above the liquid nitrogen temperature of 77 K for the first time, and the anisotropic U_eff_ was as high as 1541 cm^−1^, making it the best performing SMM to date (Figure 26a,b) [118].

**Figure 27:**
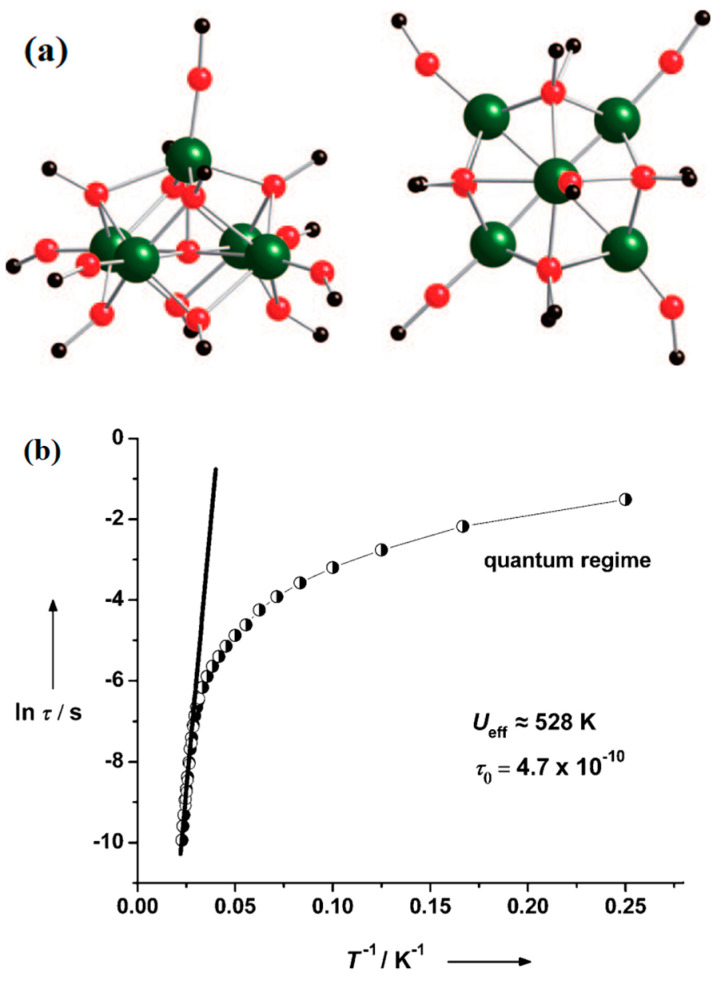
The schematic image: (**a**) Structure of [Dy_5_O(OiPr)_13_]; the left and right views represent perpendicular and parallel to the pseudofourfold axis, respectively. (Tibetan green large ball represents Dy, iPr groups trimmed for clarity). (**b**) The relationship between time (*τ*) versus T^−1^ for [Dy_5_O(OiPr)_13_] under zero static field, from data collected in frequency (●) and temperature (○) variation regime. The solid line stands for the best fit to the Arrhenius law [62].

As shown in Figure 27a, Blagg et al. [62] reported iso-propoxide-bridged Dy compounds [Dy_5_O(OiPr)_13_]. According to the Arrhenius law *τ* = *τ*_0_ exp(ΔE/k_B_ T), the relationship between ln(*τ*) versus T^−1^ (Figure 27b) could be obtained. They found that the U_eff_ of the SMMs reached approximately 530 cm^−1^.

In 2023, Luo et al. reported the preparation of [ErCl(OAr^Ad^)_3_][Na(THF)_6_] and Er(OAr^Ad^)_3_ (ArO^Ad^ = O-C_6_H_2_-2,6-Ad-4-Me). The U_eff_ arrives at 43 cm^−1^ for Er(OAr^Ad^)_3_. They found that the strong equatorial ligand field and high local symmetry are important to restrain the quantum tunneling of magnetization and realizing outstanding-performance Er-SMMs [121].

The enthusiasm for research on the synthesis of highly nucleated rare earth SMMs has been increasing because highly nucleated rare earth complexes, especially clusters, not only have nanometer dimensions but also have exotic properties not found in mononuclear rare earth complexes or low-nuclear rare earth clusters. Hong et al. synthesized spherical Dy_36_ cluster-based lattices through nicotinic acid, azide and nitrate ligands and exhibited slow magnetic relaxation behavior [122]. Tong et al. assembled Dy_11_ and Dy_12_ clusters with slow magnetic relaxation behavior by o-phenanthroline derivative ligands [123,124], and Ln-SMMs are becoming increasingly diverse.

In addition, with the development of technology, the general necessity of studying molecular spintronics [8,125], leads to the possibility of studying SMMs. This section summarizes the properties of Ln-SMM, as previously studied, including T_B_, U_eff_, and magnetic anisotropy. The summary magnetic properties of SMMs are shown in Table 3.

The U_eff_ is the main parameter to evaluate the performance of an SMM, because its height determines an important indicator of whether a SMM can be practically applied in the future, that is T_B_. Regarding the T_B_, it usually refers to the temperature at which the molecule will exhibit the magnet’s behavior. Specifically, the flip of the molecular magnetic moment slows down as the temperature decreases. When the temperature is below a critical temperature (T_B_), the molecular thermal vibration energy will not be sufficient to make the magnetization intensity (or magnetic moment) overturn the energy barrier, then the magnetic moment flip becomes blocked and tends to stay in a certain direction (the magnetization intensity can be preserved). The SMM then exhibits the behavior of a magnet.

Magnetic anisotropy plays a crucial role in the magnetic properties of SMM systems: it affects the shape of the hysteresis loop, the magnitude of the magnet coercivity, in addition to the occurrence of magnetic blocking in SMMs and the preferential orientation of the molecular magnetization.

Research in SMMs is rich and interesting, connecting not only theory and experiment, but also linking our lives and work together. The interested reader is addressed to the cited literature for more details.

## 6. Conclusions and Outlook

Rare earth SMMs are popular due to their tunable structural magnetic properties. In the nearly 30 years since the first rare earth SMMs were reported, tremendous progress has been made in single-molecule magnets, especially in single-nuclear and bi-nuclear rare earth SMMs, where some molecules have even been able to exhibit hysteresis lines above liquid nitrogen temperatures. Lanthanide elemental metal ions with a high spin ground state are good choices for the preparation of molecular materials with SMMs. In this review, the development of rare earth element monomolecular magnets with Pc as a ligand is presented, highlighting the various forms of monomolecular magnets and the current status of research on their magnetic properties, U_eff_, and T_B_. SMMs generally consist of an intrinsic metal nucleus surrounded by an organic ligand shell. With the rapid development of science and technology, great progress has been made in the study of multi-nuclear SMMs. The synthesis of multinuclear rare earth SMMs and research on magneto-structural relationships are interesting. Due to the large number of metal ions inside multinuclear rare earth SMMs, the complexity of the interactions between the metal ions increases exponentially compared to that of binuclear ones, and the properties of the SMMs can be affected. Meanwhile displaying the preparation and properties of SMMs composed of other ligands. Moreover, the development of the preparation technology of SMMs with rare earth element Pcs is summarized. Methods for regulating the magnetic anisotropy, U_eff_, and T_B_ of SMMs are also presented. Magnetic anisotropy plays a crucial role in the magnetic properties of single-molecule systems: it affects the shape of the hysteresis loop, the occurrence of magnetic blockage in single-molecule magnets and the preferential orientation of molecular magnetization. Despite significant progress has been made, some critically technical points are still needed for consideration for further applications. In general, the following strategies may provide us with a new clue to construct high-performance Ln-SMMs:

(1) Specific and detailed theoretical studies for further new straight forward synthetic strategies in production ambient conditions. One can continue the axial strong crystal modulation of the bulk field, combined with symmetry strategies to improve the rigidity of molecules and enhance intermolecular forces and magnetic exchange to synthesize higher performance SMMs with higher performance.
(2) Experimental and theoretical calculations were performed to explore more efficient methods to modulate the relaxation process to increase the T_B_ of SMMs.
(3) The synthesis of SMMs with significant anisotropy, together with a wide range of bridging ligands, has been used in the search for effective exchange interactions, and more non-centrosymmetric multinuclear SMMs with a special arrangement of metal-centered magnetic anisotropy can be designed in the synthesis work.
(4) Spintronic devices based on SMMs are an important direction of effort. It is more challenging to detect the magnetic properties of single molecule layers quickly.

All in all, we believe as scientific research progresses, more magnetic energy of rare earth SMMs can be exploited, data recording of U_eff_ and T_B_ can be enhanced again, and magnetic anisotropy can be better applied to the development of spintronics devices. This review has important implications and insights for the design of Ln-SMMs.

## Figures and Tables

**Table 1:** Crystal structure data of LnPc_2_ (Ln = Tb, Dy) [16].

	TbPc_2_	DyPc_2_
Formula	C_64_H_32_N_16_Tb	C_64_H_32_N_16_Dy
Formula weight	1183.99	1113.97
Crystal system	Orthorhombic	Orthorhombic
Space group	P2_1_2_1_2_1_(#19)	P2_1_2_1_2_1_(#19)
a (nm)	0.88	0.89
b (nm)	1.06	1.06
c (nm)	5.08	5.08
V (nm^3^)	4.76	4.76
Z	4.00	4.00
F(000)	2372.00	2268.00

**Table 2:** Summary the molecular chemical formula, main structure and their properties of most common SMMs.

Number	Molecular Chemical Formula	Rare Earth Element Category	Main Structure	Properties	Reference
1	MPc_2_ Dy, Tb	Dy, Tb	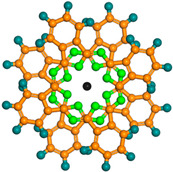	The shape of eight lobes and large magnetic anisotropy.	Refs. [15,16,17] Refs. [35,36,37,38,39] Refs. [48,49]
2	M_2_Pc_3_ (M = Dy, Tb)	Dy, Tb	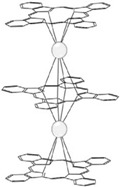	A rare earth element ion sandwiched between two Pc ligands. Large magnetic anisotropy.	Refs. [34,50]
3	[Ln_4_(acac)_4_(*μ*_2_-L)_6_(*μ*_3_-OH)_2_]·2C_2_H_5_OH (Ln = Tb and Dy)	Dy, Tb	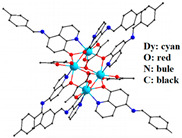	An anisotropic barrier of 82.1 K	Ref. [51]
4	[Dy_30_Co_8_Ge_12_W_108_O_408_(OH)_42_(OH_2_)_30_]^56−^	Dy	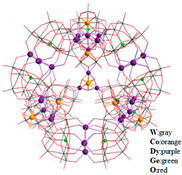	A very beautiful topology. A significant magnetic anisotropy	Ref. [65]
5	[Dy(acac)_3_(H_2_O)_2_]	Dy	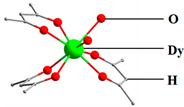	The magnetic anisotropy can be enhanced under the crystal field.	Ref. [69]
6	[LnW_10_O_36_]^9−^	Tb, Dy, Ho, Er	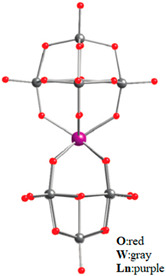	-	Ref. [71]
7	[Ln(β_2_-SiW_11_O_39_)_2_]^13−^	Tb, Dy, Ho, Er, Tm, Yb	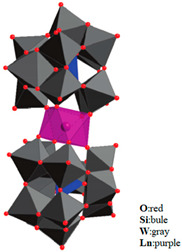	-	Ref. [72]
8	[LnP_5_W_30_O_110_]^12−^	Dy, Ho	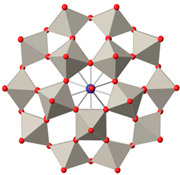	Showing magnetic hysteresis at low temper-ature and obviously big offdiagonal anisotropy parameters	Ref. [73]
9	[Dy(Cy_3_PO)_2_(H_2_O)_5_]Cl_3_·(Cy_3_PO)·H_2_O·EtOH	Dy	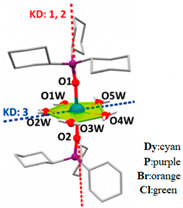	the magnetic T_B_ is 20 K	Ref. [80]

**Table 3:** Summary magnetic properties of SMMs.

Number	Performance	Definition	Typical Model	Reference
1	magnetic anisotropy	The phenomenon that the magnetism of a substance varies with the direction of the applied magnetic field is called magnetic anisotropy.	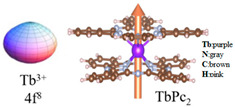	Refs. [106,111,112]
2	The effective energy barrier (U_eff_)	U_eff_ is the potential energy required for molecular magnetization (or magnetic moment) reversal).	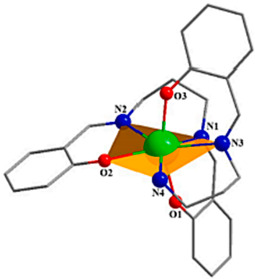	Refs. [52,55,114]
3	The blocking temperature (T_B_)	T_B_ is a key performance parameter of an SMM, one description of which refers the maximum temperature at which it is possible to observe hysteresis in the field-dependence of the magnetization, subject to the field sweep rate.	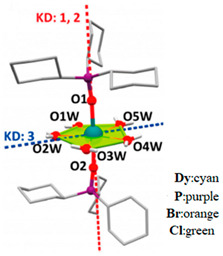	Refs. [80,96,118]

## Data Availability

No new data were created or analyzed in this study. Data sharing is not applicable to this article.

## References

[1] Sessoli R., Gatteschi D., Caneschi A. (1993). Magnetic bistability in a metal-ion cluster. Nature.

[2] Wasielewski M.R., Forbes M.D.E., Frank N.L., Kowalski K., Scholes G.D., Yuen-Zhou J., Baldo M.A., Freedman D.E., Goldsmith R.H., Goodson T. (2020). Whaley. Exploiting chemistry and molecular systems for quantum information science. Nat. Rev. Chem..

[3] Bayliss S.L., Laorenza D.W., Mintun P.J., Kovos B.D., Freedman D.E., Awschalom D.D. (2020). Optically addressable molecular spins for quantum information processing. Science.

[4] Christou G., Gatteschi D., Hendrickson D.N., Sessoli R. (2000). Single-Molecule Magnet. MRS Bull..

[5] Wang H.S., Zhang K., Song Y., Pan Z.Q. (2021). Recent advances in 3*d*-4*f* magnetic complexes with several types of non-carboxylate organic ligand. Inorg. Chim. Acta.

[6] Wang H.L., Liu T., Zhu Z.H., Peng J.M., Zou H.H., Liang F.P. (2021). A series of dysprosium clusters assembled by a substitution effect-driven out-to-in growth mechanism. Inorg. Chem. Front..

[7] Rinehart J.D., Fang M., Evans W.J., Long J.R. (2011). Strong exchange and magnetic blocking in N_2_^3−^-radical-bridged lanthanide complexes. Nat. Chem..

[8] Bogani L., Wernsdorfer W. (2008). Molecular spintronics using single-molecule magnets. Nat. Mater..

[9] Mannini M., Pineider F., Danieli C., Totti F., Sorace L., Sainctavit P., Arrio M.A., Otero E., Joly L., Cezar J.C. (2010). Quantum tunnelling of the magnetization in a monolayer of oriented single-molecule magnets. Nature.

[10] Sangregorio C., Ohm T., Paulsen C., Sesso R., Gatteschi D. (1997). Quantum Tunneling of the Magnetization in an Iron Cluster Nanomagnet. Phys. Rev. Lett..

[11] Ishikawa N., Sugita M., Ishikawa T., Koshihara S., Kaizu Y. (2003). Lanthanide Double-Decker Complexes Functioning as Magnets at the Single-Molecular Level. J. Am. Chem. Soc..

[12] Tang J.K., Hewitt I., Madhu N.T., Chastanet G., Wernsdorfer W., Anson C.E., Benelli C., Sessoli R., Powell A.K. (2006). Dysprosium Triangles Showing Single-Molecule Magnet Behavior of Thermally Excited Spin States. Angew. Chem. Int. Edit..

[13] Rinehart D.J., Fang M., Evans W.J., Long J.R. (2011). A N_2_^3−^ Radical-Bridged Terbium Complex Exhibiting Magnetic Hysteresis at 14 K. J. Am. Chem. Soc..

[14] Gatteschi D., Sessoli R. (2006). Molecular Nanomagnets.

[15] Hussain B., Savard D., Burchell T.J., Wernsdorfer W., Murugesu M. (2009). Linking high anisotropy Dy_3_ triangles to create a Dy_6_ single molecule magnet. Chem. Commun..

[16] Katoh K., Yoshida Y., Yamashita M., Miyasaka H., Breedlove B.K., Kajiwara T., Takaishi S., Ishikawa N., Isshiki H., Zhang Y.F. (2009). Direct Observation of Lanthanide(III)-Phthalocyanine Molecules on Au(111) by Using Scanning Tunneling Microscopy and Scanning Tunneling Spectroscopy and Thin-Film Field-Effect Transistor Properties of Tb(III)- and Dy(III)-Phthalocyanine Molecules. J. Am. Chem. Soc..

[17] Zhang Y.J., Wang Y.F., Liao P.L., Wang K., Huang Z.C., Liu J., Chen Q.W., Jiang J.Z., Wu K. (2018). Detection and Manipulation of Charge States for Double-Decker DyPc_2_ Molecules on Ultrathin CuO Films. ACS Nano.

[18] Avvisati G., Cardoso C., Varsano D., Ferretti A., Gargiani P., Betti M.G. (2018). Ferromagnetic and antiferromagnetic coupling of spin molecular interfaces with high thermal stability. Nano Lett..

[19] Gruber M., Ibrahim F., Boukari S., Joly L., Costa V.D., Studniarek M., Peter M., Isshiki H., Jabbar H., Davesne V. (2015). Spin-dependent hybridization between molecule and metal at room temperature through interlayer exchange coupling. Nano Lett..

[20] Javaid S., Bowen M., Boukari S., Joly L., Beaufrand J.B., Chen X., Dappe Y., Scheurer F., Kappler J.P., Arabski J. (2010). Impact on Interface Spin Polarization of Molecular Bonding to Metallic Surfaces. Phys. Rev. Lett..

[21] Ng D.K.P., Jiang J. (1997). Sandwich-type heteroleptic phthalocyaninato and porphyrinato metal complexes. Chem. Soc. Rev..

[22] Kharisov B.I., Mendes-Rokhas M.A., Ganich E.A. (2000). Traditional and electrochemical methods of synthesizing phthalocyanines and metal complexes on their base. Solvent effect. Russ. J. Coord. Chem..

[23] Nemykin V.N., Volkov S.V. (2000). Mixed-ligand complexes of lanthanides with phthalocyanine and its analogues: Synthesis, structure, and spectroscopic properties. Russ. J. Coord. Chem..

[24] Buchler J.W., Ng D.K.P., Kadish K.M., Smith K.M., Guilard R. (2000). The Porphyrin Handbook.

[25] Weiss R., Fischer J., Kadish K.M., Smith K.M., Guilard R. (2003). The Porphyrin Handbook.

[26] Kobayashi N. (2002). Dimers, trimers and oligomers of phthalocyanines and related compounds. Coord. Chem. Rev..

[27] Jiang J., Liu W., Arnold D.P. (2003). Sandwich complexes of naphthalocyanine with the rare earth metals. J. Porphyrins. Phthalocyanines.

[28] Pushkarev V.E., Tomilova L.G., Tomilov Y.V. (2008). Synthetic approaches to lanthanide complexes with tetrapyrrole type ligands. Russ. Chem. Rev..

[29] Jiang J.Z., Ng D.K.P. (2009). A Decade Journey in the Chemistry of Sandwich-Type Tetrapyrrolato-Rare Earth Complexes. Acc. Chem. Res..

[30] Rizzini A.L., Krull C., Mugarza A., Balashov T., Nistor C., Piquerel R., Klyatskaya S., Ruben M., Sheverdyaeva P.M., Moras P. (2014). Coupling of single, double, and triple-decker metal-phthalocyanine complexes to ferromagnetic and antiferromagnetic substrates. Surf. Sci..

[31] Christou G. (2005). Single-molecule magnets: A molecular approach to nanoscale magnetic materials. Polyhedron.

[32] Zhang X.H., Wang S.P. (2010). 3*d*-4*f* Single Molecule-Magnets. Prog. Chem..

[33] Gonidec M., Davies E.S., McMaster J., Amabilino D.B., Veciana J. (2010). Probing the Magnetic Properties of Three Interconvertible Redox States of a Single-Molecule Magnet with Magnetic Circular Dichroism Spectroscopy. J. Am. Chem. Soc..

[34] Hellerstedt J., Cahlik A., Svec M., de la Torre B., Moro-Lagares M., Chutora T., Papouskova B., Zoppellaro G., Mutombo P., Ruben M. (2018). On-surface structural and electronic properties of spontaneously formed Tb_2_Pc_3_ single-molecule magnets. Nanoscale.

[35] Ruan L.X., Tong J.W., Li L.R., Luo F.F., Zhang R., Qin G.W., Zhang X.M. (2020). Magnetic relaxation dependences on the central ions for Ln (Ln = Tb, Dy, Er) phthalocyanines. Appl. Phys. Lett..

[36] Thomas A.L. (1990). Phthalocyanine Research and Application.

[37] Herchel R., Zoufaly P., Nemec I. (2019). The effect of the second coordination sphere on the magnetism of [Ln(NO_3_)_3_(H_2_O)_3_]·(18-crown-6) (Ln = Dy and Er). RSC Adv..

[38] Zhang P., Guo Y.N., Tang J.K. (2013). Recent advances in dysprosium-based single-molecule magnets: Structural overview and synthetic strategies. Coord. Chem. Rev..

[39] Ishikawa N., Sugita M., Tanaka N., Ishikawa T., Koshihara S.Y., Kaizu Y. (2004). Upward Temperature Shift of the Intrinsic Phase Lag of the Magnetization of Bis (phthalocyaninato) terbiumby Ligand Oxidation Creating an S = 1/2 Spin. Inorg. Chem..

[40] Kirin I.S., Moskalev P.N., Makashev Y.A. (1965). Formation of Unusual Phthalocyanines of The Rare-Earth Elements. Russ. J. Inorg. Chem..

[41] Kirin I.S., Moskalev P.N., Makashev Y.A. (1965). Production of unusual rare earth phthalocyanines. Russ. J. Inorg. Chem..

[42] Das G.K., Zhang Y., D’Silva L., Padmanabhan P., Heng B.C., Loo J.S.C., Selvan S.T., Bhakoo K.K., Tan T.T.Y. (2011). Single-Phase Dy_2_O_3_:Tb^3+^ Nanocrystals as Dual-Modal Contrast Agent for High Field Magnetic Resonance and Optical Imaging. Chem. Mater..

[43] Eliseeva S.V., Bunzli J.C.G. (2011). Rare earths: Jewels for functional materials of the future. New J. Chem..

[44] Norek M., Kampert E., Zeitler U., Peters J.A. (2008). Tuning of the size of Dy_2_O_3_ nanoparticles for optimal performance as an MRI contrast agent. J. Am. Chem. Soc..

[45] Müller M., Montbrun R., Marz M., Fritsch V., Sürgers C., Löhneysen H.V. (2011). Switching the Conductance of Dy Nanocontacts by Magnetostr. Nano Lett..

[46] Wernsdorfer W., Aliaga-Alcalde N., Hendrickson D.N., Christou G. (2002). Exchange-biased quantum tunnelling in a supramolecular dimer of single-molecule magnets. Nature.

[47] Hill S., Edwards R., Aliaga-Alcalde N., Christou G. (2003). Quantum coherence in an exchange-coupled dimer of single-molecule magnets. Science.

[48] Martínez-Flores C., Bolívar-Pineda L.M., Basiuk V.A. (2022). Lanthanide bisphthalocyanine single-molecule magnets: A DFT survey of their geometries and electronic properties from lanthanum to lutetium. Mater. Chem. Phys..

[49] Corradini V., Candini A., Klar D., Biagi R., De Renzi V., Rizzini A.L., Cavani N., del Pennino U., Klyatskaya S., Ruben M. (2018). Probing magnetic coupling between LnPc_2_ (Ln = Tb, Er) molecules and the graphene/Ni (111) substrate with and without Au-intercalation: Role of the dipolar field. Nanoscale.

[50] Li Z.G., Gao F., Xiao Z.G., Wu X.Z., Zuo J.L., Song Y.L. (2018). Nonlinear optical properties and excited state dynamics of sandwich-type mixed (phthalocyaninato) (Schiff-base) triple-decker complexes: Effect of rare earth atom. Opt. Laser. Technol..

[51] Wang B., Wei C.Y. (2020). Structures fluorescent properties and single-molecule-magnet behavior of two Ln_4_ (Ln^III^ = Tb and Dy) clusters. J. Mol. Struct..

[52] Wang H.T., Pu J.R., Li X., Zhang Y., Zhang Y., Li L., Fang M. (2021). A novel Dy_4_ cluster constructed by an 8 hydroxyquinoline Schiff base showing remarkable single molecule magnet behavior. Polyhedron.

[53] Shi X.H., Wang W.M., Yan L.L., Fan C.J., Pang J.L., Wu Z.L. (2021). Crystal structure and single-molecule magnet behavior of a novel tetranuclear Dy(III)-based cluster. J. Mol. Struct..

[54] Wu J.J., Li X.L., Droitte L.L., Cador O., Guennic B.L., Tang J.K. (2021). Coordination anion effects on the geometry and magnetic interaction of binuclear Dy_2_ single-molecule magnets. Dalton. Trans..

[55] Canaj A.B., Dey S., Wilson C., Céspedes O., Rajaraman G., Murrie M. (2020). Engineering macrocyclic high performance pentagonal bipyramidal Dy(iii) single-ion magnets. Chem. Commun..

[56] Zhu Z.H., Guo M., Li X.L., Tang J.K. (2019). Molecular magnetism of lanthanide: Advances and perspectives. Coord. Chem. Rev..

[57] Yang C., Lei W.T., Xin X.Y., Qiao N., Hao F.F., Zhang Q.F., Zhou Y., Fang M., Wang W.M. (2022). Construction of two Ln(III)_2_ (Ln = Dy and Er) compounds by a polydentate Schiff-based ligand: Structure and remarkable single-molecule magnet behavior. J. Mol. Struct..

[58] Wang H.T., Niu X.Y., Zhang G.X., Jiao Y.H., Wu J.Y., Zhang Y., Hou Y.L. (2022). Two butterfly-shaped Ln^III^_2_ compounds constructed by a multidentate Schiff base ligand: Structures, fluorescence properties and SMMs behaviors. Inorg. Chem. Commun..

[59] Qiao N., Li X.X., Chen Y., Xin X.Y., Yang C., Dong S.S., Wang Y.Z., Li X.J., Hua Y.P., Wang W.M. (2022). Three Ln_2_ compounds (Gd_2_, Tb_2_ and Dy_2_) with a Ln_2_O_2_ center showing magnetic refrigeration property and single-molecular magnet behavior. Polyhedron.

[60] Borah A., Murugavel R. (2022). Magnetic relaxation in single-ion magnets formed by less-studied lanthanide ions Ce(III), Nd(III), Gd(III), Ho(III), Tm(II/III) and Yb(III). Coordin. Chem. Rev..

[61] Sun H., Guo Y., Cui Y.F., Li D.W., Yang G.C., She Y.Y., Zhang Q., Li Y.H., Zhang Y.Q., Yao J.L. (2023). Dy_3_ and Gd_3_ Complexes with Dy_3_ Exhibiting Field-Induced Single-Molecule Magnet Behaviour. J. Mol. Struct..

[62] Blagg R.J., Muryn C.A., McInnes E.J., Tuna F., Winpenny R.E. (2011). Single Pyramid Magnets: Dy_5_ Pyramids with Slow Magnetic Relaxation to 40 K. Angew. Chem. Int. Ed..

[63] Blagg R.J., Ungur L., Tuna F., Speak J., Comar P., Collison D., Wernsdorfer W., McInnes E.J.L., Chibotaru L.F., Winpenny R.E.P. (2013). Magnetic Relaxation Pathways in Lanthanide Single-Molecule Magnets. Nat. Chem..

[64] Langley S.K., Wielechowski D.P., Vieru V., Chilton N.F., Chibotaru L.F., Murray K.S. (2015). The first 4*d*/4*f* single-molecule magnet containing a {Ru^III^_2_Dy^III^_2_} core. Chem. Commun..

[65] Ibrahim M., Mereacre V., Leblanc N., Wernsdorfer W., Anson C.E., Powell A.K. (2015). Self-Assembly of a Giant Tetrahedral 3*d*-4*f* Single-Molecule Magnet within a Polyoxometalate System. Angew. Chem. Int. Ed..

[66] Rinehart J.D., Long J.R. (2011). Exploiting single-ion anisotropy in the design of f-element single-molecule magnets. Chem. Sci..

[67] Chilton N.F., Collison D., McInnes E.J.L., Winpenny R.E.P., Soncini A. (2013). An electrostatic model for the determination of magnetic anisotropy in dysprosium complexes. Nat. Commun..

[68] Jiang S.D., Qin S.X. (2015). Prediction of the quantized axis of rare-earth ions: The electrostatic model with displaced point charges. Inorg. Chem. Front..

[69] Jiang S.D., Wang B.W., Su G., Wang Z.M., Gao S. (2010). A Mononuclear Dysprosium Complex Featuring Single-Molecule Magnet Behavior. Angew. Chem..

[70] Gao C., Yang Q., Wang B.W., Wang Z.M., Gao S. (2016). Evaporable lanthanide single-ion magnet. CrystEngComm.

[71] AlDamen M.A., Clemente-Juan J.M., Coronado E., Martı C., Gaita-Arinõ A. (2008). Mononuclear Lanthanide Single-Molecule Magnets Based on Polyoxometalates. J. Am. Chem. Soc..

[72] AlDamen M.A., Cardona-Serra S., Clemente-Juan J.M., Coronado E., Gaita-Arin A., Martı C., Luis F., Montero O. (2009). Mononuclear Lanthanide Single-Molecule Magnets Based on the Polyoxometalates [Ln(W_5_O_18_)_2_]^9−^ and [Ln(β_2_-SiW_11_O_39)2_]^13−^ (Ln^III^ = Tb, Dy, Ho, Er, Tm, and Yb). Inorg. Chem..

[73] Cardona-Serra S., Clemente-Juan J.M., Coronado E., Gaita-Arino A., Camon A., Evangelisti M., Luis F., Martinez-Perez M.J., Sese J. (2012). Lanthanoid Single-Ion Magnets Based on Polyoxometalates with a 5-fold Symmetry: The Series [LnP_5_W_30_O_110_]^12−^(Ln^3+^ = Tb, Dy, Ho, Er, Tm, and Yb). J. Am. Chem. Soc..

[74] Dey A., Kalita P., Chandrasekhar V. (2018). Lanthanide(III)-Based Single-Ion Magnets. ACS Omega.

[75] Ding Y.S., Han T., Zhai Y.Q., Reta D., Chilton N.F., Winpenny R.E.P., Zheng Y.Z. (2020). A Study of Magnetic Relaxation in Dysprosium(III) Single-Molecule Magnets. Chem. Eur. J..

[76] Ma Y., Zhai Y.Q., Ding Y.S., Han T., Zheng Y.Z. (2020). Understanding a Pentagonal-Bipyramidal Holmium(III) Complex with Record Energy Barrier for Magnetisation Reversal. Chem. Commun..

[77] Huang G.Z., Ruan Z.Y., Zheng J.Y., Chen Y.C., Wu S.G., Liu J.L., Tong M.L. (2020). Seeking magneto-structural correlations in easily tailored pentagonal bipyramid Dy(III) single-ion magnets. Sci. China Chem..

[78] Yu K.X., Kragskow J.G.C., Ding Y.S., Zhai Y.Q., Reta D., Chilton N.F., Zheng Y.Z. (2020). Enhancing Magnetic Hysteresis in Single-Molecule Magnets by Ligand Functionalization. Chem.

[79] Ding Y.S., Chilton N.F., Winpenny R.E.P., Zheng Y.Z. (2016). On approaching the limit of molecular magnetic anisotropy: A near-perfect pentagonal bipyramidal Dysprosium(III) single-molecule magnet. Angew. Chem. Int. Ed..

[80] Chen Y.C., Liu J.L., Ungur L., Liu J., Li Q.W., Wang L.F., Ni Z.P., Chen X.M., Tong M.L. (2016). Symmetry-supported magnetic blocking at 20 K in pentagonal bipyramidal Dy(III) single-ion magnets. J. Am. Chem. Soc..

[81] Yuan Z.D., Qin Y.R., Sha J.Q., Wang Y.Y., Zhang H.F. (2022). Two single-molecule magnets {Dy_4_O_8_} based on mixed ligand strategy. Inorg. Chem. Commun..

[82] Pushkarev V.E., Tomilova L.G., Nemykin V.N. (2016). Historic overview and new developments in synthetic methods for preparation of the rare earth tetrapyrrolic complexes, Coordin. Chem. Rev..

[83] Dubinina T.V., Paramonova K.V., Trashin S.A., Borisova N.E., Tomilova L.G., Zefirov N.S. (2014). Novel near-IR absorbing phenyl-substituted phthalo- and naphthalocyanine complexes of lanthanide(III): Synthesis and spectral and electrochemical properties. Dalton. Trans..

[84] Kirin I.S., Moskalev P.N., Ivannikova N.V. (1967). Preparation and Properties of Neodymium Phthalocyanine. Russ. J. Inorg. Chem..

[85] Kasuga K., Ando M., Morimoto H., Isa K. (1986). Preparation of new phthalocyanine complexes of yttrium (iii) and some lanthanoid (iii) ions. Chem. Lett..

[86] Gonidec M., Biagi R., Corradini V., Moro F., Renzi V.D., Pennino U.D., Summa D., Muccioli L., Zannoni C., Amabilino D.B. (2011). Surface Supramolecular Organization of a Terbium(III) Double-Decker Complex on Graphite and its Single Molecule Magnet Behavior. J. Am. Chem. Soc..

[87] Sokolova T.N., Lomova T.N., Morozov V.V., Berezin B.D. (1994). Complex Compounds of Lanthanides with Phthalocyanine: Double Sandwich. Koord. Khim..

[88] Sadak M.M., Roncali J., Garnier F. (1986). Lanthanides-Phthalocyanines complexes: From a diphthalocyanine Pc_2_Ln to a super complex Pc_3_Ln_2_. J. Chim. Phys..

[89] Ruan L.X., Tong J.W., Qin G.W., Zhou L.Q., Jiao X.C., Zhang X.M. (2020). Magnetic Modification and the Mechanism of Tb-Phthalocyanine Single-Molecule Magnets Prepared by a High Yield Method. Eur. J. Inorg. Chem..

[90] Zhang P., Benner F., Chilton N.F., Demir S. (2022). Organometallic lanthanide bismuth cluster single-molecule magnets. Chemistry.

[91] Lux F., Graw F., Graw D. (1968). Diphthalocyaninato-thorium (Ⅳ) and-uranium(Ⅳ). Angew. Chem. Int. Ed. Eng..

[92] Buchler J.W., Kappelmann H.G., Knoff M., Lay K.L., Pfeifer S. (1983). Metallkomplexe mit Tetrapyrrol-Liganden, XXXI[1] Neutral and anionoid Bisporphinates des Cers und Praseodyms. Z. Nat. B.

[93] Li Z.H., Luo Q.C., Zheng Y.Z. (2021). Recent Progress of Rare-Earth Single-Molecule Magnets. J. Chin. Soc. Rare Earths.

[94] Ungur L. (2018). Introduction to the electronic structure, luminescence, and magnetism of lanthanides. Lanthanide-Based Multifunctional Materials.

[95] Meng Y.S., Jiang S.D., Wang B.W., Gao S. (2016). Understanding the Magnetic Anisotropy toward Single-Ion Magnets. Acc. Chem. Res..

[96] Wernsdorfer W., Sessoli R. (1999). Quantum phase interference and parity effects in magnetic molecula culsters. Science.

[97] Cinchetti M., Dediu V.A., Hueso L.E. (2017). Activating the molecular spinterface. Nat. Mater..

[98] Sun M., Mi W. (2018). Progress in organic molecular/ferromagnet spinterfaces: Towards molecular spintronics. J. Mater. Chem C.

[99] Tran T.L.A., Çakır D., Wong P.K.J., Preobrajenski A.B., Brocks G., van der Wiel W.G., de Jong M.P. (2013). Magnetic Properties of bcc-Fe(001)/C_60_ Interfaces for Organic Spintronic. ACS Appl. Mater. Interfaces.

[100] Sanvito S. (2010). Molecular spintronics: The rise of spinterface science. Nat. Phys..

[101] Atodiresei N., Brede J., Lazi P., Caciuc V., Hoffmann G., Wiesendanger R., Blügel S. (2010). Design of the Local Spin Polarization at the Organic-Ferromagnetic Interface. Phys. Rev. Lett..

[102] Han X., Mi W., Wang X. (2019). Spin polarization and magnetic properties at the C_60_/Fe_4_N(001) spinterface. J. Mater. Chem. C.

[103] Han X., Mi W., Wang D. (2020). Tunneling magnetoresistance and light modulation in Fe_4_N(La_2_/_3_Sr_1/3_MnO_3_)/C_60_/Fe_4_N single-molecule magnetic tunnel junctions. J. Mater. Chem. C.

[104] Liu J.L., Chen Y.C., Tong M.L. (2018). Symmetry strategies for high performance lanthanide-based single-molecule magnets. Chem. Soc. Rev..

[105] Bernot K., Luzon J., Bogani L., Etienne M., Sangregorio C., Shanmugam M., Caneschi A., Sessoli R., Gatteschi D. (2009). Magnetic Anisotropy of Dysprosium(III) in a Low-Symmetry Environment: A Theoretical and Experimental Investigation. J. Am. Chem. Soc..

[106] Zhu M.M., Pan H.D., Teng Q.H., Liang F.P., Wang K. (2023). Slow magnetic relaxation behavior of a {Dy_2_} complex based on a large π-conjugated bridging ligand. Polyhedron.

[107] Wang M.M., Meng X.X., Song F., He Y.F., Shi W., Gao H.L., Tang J.K., Peng C. (2018). Reversible structural transformation induced switchable single-molecule magnet behavior in lanthanide metal-organic frameworks. Chem. Commun..

[108] Cen P.P., Liu X.Y., Soria J.F., Zhang Y.Q., Xie G., Chen S.P., Pardo E. (2019). Capping Ndonor ligands modulate the magnetic dynamics of DyIII β-diketonate single-ion magnets with D4d symmetry. Chem. Eur. J..

[109] Luzon J., Bernot K., Hewitt I.J., Anson C.E., Powell A.K., Sessoli R. (2008). Spin Chirality in a Molecular Dysprosium Triangle: The Archetype of the Noncollinear Ising Model. Phys. Rev. Lett..

[110] Lin P.H., Burchell T.J., Ungur L., Chibotaru L.F., Wernsdorfer W., Murugesu M. (2009). A Polynuclear Lanthanide Single-Molecule Magnet with a Record Anisotropic Barrier. Angew. Chem..

[111] Ruan L.X., Tong J.W., Luo F.F., Wu Y.Z., Qin G.W., Jiao X.C., Zhang X.M. (2022). The magnetic anisotropy of Tb-phthalocyanine films effected by molecular orientation. Appl. Surf. Sci..

[112] Zhang W.B., Muhtadi A., Iwahara N., Ungur L., Chibotar L.F. (2020). Magnetic anisotropy in divalent lanthanide compounds. Angew. Chem. Int. Ed..

[113] Guo Y.N., Xu G.F., Gamez P., Zhao L., Lin S.Y., Deng R., Tang J., Zhang H.J. (2010). Two-Step Relaxation in a Linear Tetranuclear Dysprosium(III) Aggregate Showing Single-Molecule Magnet Behavior. J. Am. Chem. Soc..

[114] Chen C.P., Wang Y.F., Qin P., Zou H.H., Liang F.P. (2023). A Dy^III^ single-ion magnet with D_5h_ configuration. Inorg. Chim. Acta.

[115] Hu J., Wu R.Q. (2013). Control of the Magnetism and Magnetic Anisotropy of a Single-Molecule Magnet with an Electric Field. Phys. Rev. Lett..

[116] Tsukahara N., Noto K.I., Ohara M., Shiraki S., Takagi N., Takata Y., Miyawaki J., Taguchi M., Chainani A., Shin S. (2009). Adsorption-Induced Switching of Magnetic Anisotropy in a Single Iron(II) Phthalocyanine Molecule on an Oxidized Cu(110) Surface. Phys. Rev. Lett..

[117] Goodwin C.A.P., Ortu F., Reta D., Chilton N.F., Mills D.P. (2017). Molecular magnetic hysteresis at 60 kelvin in dysprosocenium. Nature.

[118] Guo F.S., Day B.M., Chen Y.C., Tong M.L., Mansikkamaki A., Layfield R.A. (2018). Magnetic hysteresis up to 80 kelvin in a dysprosium metallocene single-molecule magnet. Science.

[119] Leuenberger M.N., Loss D. (2001). Quantum computing in molecular magnets. Nature.

[120] Kilpatrick A.F.R., Guo F.S., Day B.M., Mansikkamaki A., Layfield R.A., Cloke F.G.N. (2018). Single-molecule magnet properties of a monometallic dysprosium pentalene complex. Chem. Commun..

[121] Luo Q.C., Ge N., Zhai Y.Q., Wang T.B., Sun L., Sun Q., Li F.N., Fu Z.D., Zheng Y.Z. (2023). Switching the coordination geometry to enhance erbium(III) single-molecule magnets Chinese. Chem. Lett..

[122] Miao Y.L., Jiang F., Kong X., Yuan D., Long L., Al-Thabaiti S.A., Hong M. (2013). Two polymeric 36-metal pure lanthanide nanosize clusters. Chem. Sci..

[123] Miao Y.L., Li J.Y., Leng J.D., Ou Y.C., Tong M.L. (2011). Two novel Dy_8_ and Dy_11_ clusters with cubane [Dy_4_(μ_3_-OH)_4_]^8+^ units exhibiting slow magnetic relaxation behaviour. Dalton. Trans..

[124] Miao Y.L., Liu J.L., Leng J.D., Lin Z.J., Tong M.L. (2011). Chloride templated formation of {Dy_12_(OH)_16_}^20+^ cluster core incorporating 1,10-phenanthroline-2,9-dicarboxylate. CrystEngComm.

[125] Coronado E. (2020). Molecular magnetism: From chemical design to spin control in molecules, materials and devices. Nat. Rev. Mater..

